# Faradaic Pixels for Precise Hydrogen Peroxide Delivery to Control M‐Type Voltage‐Gated Potassium Channels

**DOI:** 10.1002/advs.202103132

**Published:** 2021-11-26

**Authors:** Oliya S. Abdullaeva, Ihor Sahalianov, Malin Silverå Ejneby, Marie Jakešová, Igor Zozoulenko, Sara I. Liin, Eric Daniel Głowacki

**Affiliations:** ^1^ Laboratory of Organic Electronics ITN Campus Norrköping Linköping University Norrköping SE‐60174 Sweden; ^2^ Wallenberg Center for Molecular Medicine Linköping University Linköping SE‐58185 Sweden; ^3^ Bioelectronics Materials and Devices Lab Central European Institute of Technology Brno University of Technology Purkyňova 123 Brno 61200 Czech Republic; ^4^ Department of Biomedical and Clinical Sciences Linköping University Linköping SE‐58185 Sweden

**Keywords:** electrochemistry, organic bioelectronics, potassium channels, reactive oxygen species, *Xenopus laevis* oocytes

## Abstract

H_2_O_2_ plays a significant role in a range of physiological processes where it performs vital tasks in redox signaling. The sensitivity of many biological pathways to H_2_O_2_ opens up a unique direction in the development of bioelectronics devices to control levels of reactive‐oxygen species (ROS). Here a microfabricated ROS modulation device that relies on controlled faradaic reactions is presented. A concentric pixel arrangement of a peroxide‐evolving cathode surrounded by an anode ring which decomposes the peroxide, resulting in localized peroxide delivery is reported. The conducting polymer (poly(3,4‐ethylenedioxythiophene) (PEDOT), is exploited as the cathode. PEDOT selectively catalyzes the oxygen reduction reaction resulting in the production of hydrogen peroxide (H_2_O_2_). Using electrochemical and optical assays, combined with modeling, the performance of the devices is benchmarked. The concentric pixels generate tunable gradients of peroxide and oxygen concentrations. The faradaic devices are prototyped by modulating human H_2_O_2_‐sensitive Kv7.2/7.3 (M‐type) channels expressed in a single‐cell model (*Xenopus laevis* oocytes). The Kv7 ion channel family is responsible for regulating neuronal excitability in the heart, brain, and smooth muscles, making it an ideal platform for faradaic ROS stimulation. The results demonstrate the potential of PEDOT to act as an H_2_O_2_ delivery system, paving the way to ROS‐based organic bioelectronics.

## Graphical abstract

Microfabricated electrochemical devices that precisely control oxygen and hydrogen peroxide gradients for biological experiments are presented.

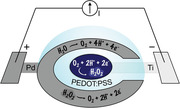

## 1 Introduction

Reactive oxygen species (ROS) regulate vital biological processes and are involved in essential signaling pathways during redox metabolism.^[^ ^1^, ^2^, ^3^, ^4^ ^]^ While the majority of studies are dedicated to understanding their toxicity in connection with numerous medical conditions, for example, neurodegenerative diseases, atherosclerosis, or aging,^[^ ^5^ ^]^ an increasing number of reports support the notion that ROS exhibit important physiological roles in maintaining redox homeostasis.^[^ ^6^, ^7^, ^8^, ^9^ ^]^ Especially with regard to hydrogen peroxide (H_2_O_2_) the research field of redox biology has gained valuable insight into ROS‐mediated mechanisms that underlie a variety of functions ranging from immune response to angiogenesis.^[^ ^5^, ^6^, ^10^ ^]^ As early as 1970, pioneering work by Sies & Chance provided the first evidence that hydrogen peroxide is generated in aerobic biological cells.^[^ ^11^, ^12^ ^]^ So far, several intracellular sites have been identified as physiological sources of H_2_O_2_, most notably the mitochondria and NADPH oxidase.^[^ ^13^ ^]^ Moreover, it is established that H_2_O_2_ is produced during oxidative protein folding and via peroxisomal enzymes.^[^ ^13^ ^]^ The most prominent role of H_2_O_2_ is known to be its contribution to redox signaling as a secondary messenger.^[^ ^14^ ^]^ The non‐radical nature and longer half‐life under physiological conditions allow H_2_O_2_ to act as a signaling molecule.^[^ ^15^, ^16^ ^]^ The reversible oxidation of thiol groups belonging to deprotonated cysteines residues in proteins stands out among the several multifaceted redox reactions that can be initiated by H_2_O_2_ which in turn results in altered protein activity.^[^ ^17^ ^]^ These signaling mechanisms have a major impact on cell proliferation, survival, and differentiation. These effects can proceed with even low H_2_O_2_ concentrations in the range of 1–10 nm.^[^ ^6^, ^18^, ^19^, ^20^ ^]^ Here, the fine redox balance is maintained via scavenging systems (e.g., catalase) that remove excess amounts of ROS which otherwise would cause irreversible damage to nucleic acids, proteins, and lipids.^[^ ^21^ ^]^

In the past decades, H_2_O_2_ has also been gradually coming to the fore as an oxidative modulator of ion channels. Several more recent electrophysiological studies have revealed the ability of H_2_O_2_ to directly activate ion channels, including ATP‐sensitive potassium (K_ATP_) and transient receptor potential (TRP) channels.^[^ ^22^ ^]^ In particular, the sensitivity of voltage‐gated Kv7.2/7.3 channels toward oxidative modification has gained much attention since this type of potassium channel exerts crucial tasks related to neuronal activity.^[^ ^22^, ^23^ ^]^ Heterotetrameric Kv7.2/7.3 channels are encoded by *KCNQ2* and *KCNQ3* genes and control neuronal excitability in hippocampal as well as in dorsal root ganglion neurons by increasing the threshold for action potential firing.^[^ ^24^, ^25^, ^26^ ^]^ The anti‐excitable role of Kv7.2/7.3 channels is largely due to the fact that subthreshold membrane voltages (≈−60 mV) are sufficient to activate Kv7.2/7.3, and that Kv7.2/7.3 channels remain in their activated state despite extended time of activation.^[^ ^27^, ^28^ ^]^ Consequently, Kv7.2/7.3 channels generate an outward potassium current, also referred to as M‐current, which contributes to the negative resting membrane potential of neurons. M‐current constitutes a neuroprotective mechanism that prevents uncontrollable excess action potential firing.^[^ ^27^, ^28^, ^29^ ^]^ The physiological relevance of Kv7.2/7.3 becomes apparent in neurological conditions where their regulatory functions are lost due to inherited mutations, for instance in epilepsy.^[^ ^30^ ^]^ Malfunction and consequently loss of function of these ion channels leads to a pathological increase in neuronal excitability.^[^ ^26^, ^30^ ^]^ Neuropathic pain, commonly observed in cancer patients, is a further condition that is being associated with impaired Kv7.2/7.3 function and hyperexcitability.^[^ ^31^, ^32^, ^33^ ^]^ According to more recent work by Gamper et al. physiological concentrations of H_2_O_2_ can activate Kv7.2/7.3 and enhance M‐currents, making it a potentially potent therapeutic with a desired anti‐excitable effect/silencing effect.^[^ ^23^ ^]^ For this reason, we have chosen this type of ion channel as a target for faradaic H_2_O_2_‐based stimulation in this work.

The role of H_2_O_2_ in mediating (electro)physiological pathways encouraged us to develop a method for controlled electrical delivery of H_2_O_2_ in physiological environment. In just the past few years, a nascent field of faradaic delivery concepts for neuromodulation has begun to emerge. Recently, Tian and colleagues reported a photoactivated silicon/Au nanowire system for intracellular H_2_O_2_ delivery,^[^ ^34^ ^]^ suggesting this as a potent approach to enable novel experimental procedures to understand ROS effects. In pioneering works from Antognazza and coworkers, the photochemical production of peroxide/ROS by organic semiconductors, namely polythiophenes, was demonstrated at the level of several biological targets: charge‐transfer to redox proteins,^[^ ^35^ ^]^ stimulation of transient‐receptor vanilloid subtype ion channels,^[^ ^36^ ^]^ and ultimately control of stem cell differentiation.^[^ ^37^ ^]^ Nitric oxide (NO) is another example of a redox signaling molecule, which was recently exploited by Park and colleagues^[^ ^38^ ^]^ in a device for in vivo electrochemical NO delivery. On the other hand, electrochemical devices for manipulation of O_2_/H_2_O_2_ for biological applications have not been developed to date.

This paper reports a method to precisely control electrical dosing of peroxide levels. The starting point for our device is the conducting polymer (poly(3,4‐ethylenedioxythiophene) (PEDOT). Recently, it was shown that PEDOT can efficiently mediate the reduction of oxygen to H_2_O_2_ following a two‐electron oxygen reduction reaction mechanism.^[^ ^39^ ^]^ In the present study, the working electrode consists of a circular shaped PEDOT thin film that is enclosed by a palladium counter electrode that functions as a confining barrier for the H_2_O_2_ generated by the central PEDOT pixel. This device configuration offers localized, on‐demand production of H_2_O_2_. Through the experiments and we have carried out, it becomes apparent that there is a fine interplay between peroxide and oxygen levels. We validate the physiological effect of electrochemically‐delivered H_2_O_2_ on Kv7.2/7.3 channels expressed in *Xenopus laevis* oocytes. The oocytes are large and robust cells that allow us to test electrical peroxide delivery in a proof‐of‐concept fashion without confounding variables present in more complex cells. The enhancement of potassium M‐currents during two‐electrode voltage‐clamp experiments validates the ability of the faradaic pixel to modulate ion channel activity via local dosing of peroxide. This work highlights the potential of PEDOT to act as faradaic delivery material for modulation of neuronal voltage‐gated ion channels and thus takes a step forward in understanding and establishing faradaic stimulation mechanisms which will constitute the basis for future advancement of ROS‐mediated neuromodulation.

## 2 Results and Discussion

### 2.1 Electrochemistry of Faradaic Pixels: The Balance between Oxygen and Hydrogen Peroxide

**Figure 1:**
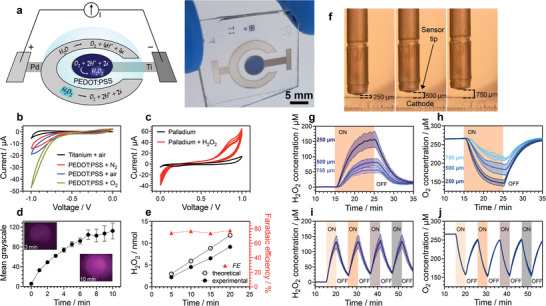
Faradaic pixels electrically modulate hydrogen peroxide and oxygen concentrations. a) Schematic and photo of the device, featuring a circular PEDOT:PSS cathode in the center, surrounded by a palladium anode. The cathode produces H_2_O_2_ via the 2‐electron oxygen reduction reaction, while the anode completes the DC electrochemical circuit by the anodic reactions of water oxidation and peroxide oxidation. b) Cyclic voltammetry recordings of PEDOT:PSS versus bare Titanium in pH 7.4 electrolyte with different O_2_ contents: air saturated, 0% (purged with N_2_), and 100% (purged with O_2_). The volume of solution that was placed on top of the active area (6.15 mm^2^) was 30 µL. Larger volumes were initially purged with either O_2_ or N_2_ and subsequently an aliquot of 30 µL used for recordings. c) Cyclic voltammetry (4 cycles) measurement of the palladium counter electrode before and after addition of 10 mm H_2_O_2_. d) Fluorescent images of the electrolyte droplet containing Amplex UltraRed reagent placed on top of the PEDOT:PSS pixel. The PEDOT:PSS pixel device was operated for 10 min and changes in the fluorescent signal of the Amplex UltraRed reagent were recorded over time. Increase in fluorescence intensity (greyscale) was evaluated by plotting the intensity over time. e) Mean quantities (nmol, left axis) of H_2_O_2_ produced by the device obtained via the HRP‐TMB assay after running the PEDOT faradaic pixel device galvanostatically at 10 µA cm^−2^ for 5, 10, 15 and 20 min (± SD, *n* = 3–5, number of measured samples in total = 5). Faradaic efficiency is calculated at each time point, and is plotted on the right axis. Threoretical values indicate a situation with 100% faradaic yield. f) Digital camera imaging was used to calculate the distance, *d =* 250, 500, 750 µm, of the amperometric sensor from the surface of the pixel. Distance was calibrated using the known thickness of the microscope slide as a standard. g,h) mean H_2_O_2_ and O_2_ concentration traces acquired via local amperometric recordings using H_2_O_2_ and O_2_ sensors 250, 500, and 750 µm above PEDOT:PSS film surface for a run time of 10 min. i,j) mean H_2_O_2_ and O_2_ concentration traces measured 250 µm above the device surface during alternating on/off time periods with a duration of 5 min (on)/5.35 min (off), respectively (± SD, *n* = 3–6, number of measured samples in total: 4).

The faradaic peroxide delivery devices were designed using a concentric arrangement of a central cathode surrounded by an anode ring (**Figure** **1**a). This way, H_2_O_2_ produced at the cathode will be spatially confined by the anode, as H_2_O_2_ which arrives at the anode will be readily oxidized back to O_2_. It is critical to choose suitable cathode and anode materials. The first consideration is to select a cathode that reduces oxygen selectively to H_2_O_2_. The cathodic electrochemical reactions relevant in this work are shown in the following:

(1) $$O_{2}+2e^{-}+2H^{+}\to H_{2}O_{2}$$

(2) $$O_{2}+4H^{+}+4e^{-}\to 2H_{2}O$$

(3) $$H_{2}O_{2}+2e^{-}+2H^{+}\to 2H_{2}O$$

(4) $$2H^{+}+2e^{-}\to H_{2}$$

An ideal electrocatalyst for this application is one that enables Equation (1), while not favoring the remaining Equations (2)–(4). For this purpose we use the conducting polymer formulation PEDOT with poly(styrene sulfonate), shortened as PEDOT:PSS, or just PEDOT in this paper. PEDOT is known to be a redox mediator for selective two‐electron reduction of oxygen to hydrogen peroxide (Equation (1)).^[^ ^39^ ^]^ Remarkably, compared to other 2‐electron catalysts, PEDOT allows for the net accumulation of peroxide because the further reduction (Equation (3)) is kinetically hindered. The four‐electron oxygen reduction reaction (Equation (2)) does not occur at any observable rate. The two‐electron hydrogen‐evolution reaction (Equation (4)) can happen on PEDOT, however only at very low pH values.^[^ ^40^ ^]^ This reaction is not efficient at pH values exceeding 3. Therefore, under neutral pH conditions, Equation (1) can be expected to predominate on PEDOT. Facile processability and good biocompatibility make PEDOT a logical choice for the peroxide‐evolving cathode element in this device.

The anode material should satisfy the conditions of good stability under positive polarization, insensitivity to corrosion by peroxide, and the ability to catalyze both peroxide oxidation (Equation (5)) and water oxidation (Equation (6)):

(5) $$H_{2}O_{2}\to O_{2}+2H^{+}+2e^{-}$$

(6) $$2H_{2}O\to O_{2}+4H^{+}+4e^{-}$$

(7) $$2H_{2}O_{2}\to O_{2}+2H_{2}O$$

These conditions are met by many of the group VIII transition elements, the so‐called platinum‐like metals such as palladium, platinum, ruthenium, or iridium.^[^ ^41^ ^]^ Based on easy processability, high stability, and relatively lowest cost, we chose palladium as the anodic electrode.^[^ ^42^ ^]^ Another property of the platinum metals is that they efficiently catalyze the nonfaradaic disproportionation reaction of hydrogen peroxide to water and oxygen (Equation (7)).^[^ ^43^ ^]^ This is an important aspect of the faradaic pixel design, as this means that the palladium ring will decompose peroxide regardless of whether current is on or off.

The complete design of the faradaic pixel therefore consists of a central PEDOT cathode, surrounded by a palladium ring (Figure 1a). We used titanium as a suitable underlying conductor for both cathode and anode. Titanium is chosen due to its relative electrochemical inertness and good adhesion with PEDOT and palladium. All conductive leads in the layout are passivated using parylene‐c encapsulation, which is patterned using conventional photolithography methods (see Experimental Section, and Figure S1, Supporting Information). Anticipating conditions necessary for the Kv7.2/7.3 channel measurements discussed in Section 2.3, all experiments are carried out in a physiological buffer solution (1K Solution, see Experimental Section), with relatively high Na^+^ and low K^+^ concentrations, and a pH of 7.4. Using cyclic voltammetry (CV), we probed the electrochemical properties of the cathode (Figure 1b) and anode (Figure 1c). In particular, it was necessary to verify the cathodic reduction of oxygen to peroxide (Equation (1)), and the oxidation of peroxide back to oxygen (Equation (5)). It is important to consider that under these experimental conditions, oxygen will be the limiting reagent. While the selectivity of oxygen reduction to peroxide on PEDOT electrodes has been characterized in the literature,^[^ ^39^, ^44^ ^]^ results were reported under pure 1 atm O_2_. In this work, we are concerned with oxygen reduction to peroxide under ambient 0.21 atm O_2_ conditions. This is critical since the nonpolar dioxygen molecule has low solubility in water. The highest equilibrium concentration of oxygen in water under 1 atm of pure O_2_ is around 2 mm. Under ambient conditions in physiological media, [O_2_] is around 200 to 300 µm. Oxygen is therefore the limiting reagent in the cathodic reduction of O_2_ to H_2_O_2_. The effects of oxygen concentration are readily apparent in CVs of PEDOT cathodes in physiological buffer solution, pH 7.4 (Figure 1b). Comparing deoxygenated solution (N_2_‐purged), ambient air, and 1 atm O_2_ bubbled solutions shows the clear rise in cathodic current as the amount of oxygen is increased. This evidences the presence of the oxygen reduction reaction as the primary cathodic process, with an onset potential of ≈−0.5 V versus Ag/AgCl. Measurement of a bare titanium cathode in air, in contrast, demonstrates a near complete absence of cathodic currents relative to PEDOT‐coated titanium.

The palladium electrode CV (Figure 1c) demonstrated the expected anodic water splitting behavior (Equation (6)). Upon addition of H_2_O_2_ to the electrolyte, pronounced peroxide oxidation (Equation (5)) and peroxide reduction (Equation (3)) peaks appear. This indicates that palladium can fulfill the role of anodic counter electrode, supporting water and peroxide oxidation. Based on these CV experiments, the PEDOT electrode is therefore a suitable cathode for sustained peroxide evolution, and palladium is an ideal counter electrode that will close the redox cycle and consume peroxide.

We next verified that the cathodic oxygen reduction reaction on PEDOT was indeed peroxide evolution (Equation (1)). A convenient qualitative visualization is the use of Amplex Red, a fluorescent reporter for H_2_O_2_ presence. We used this fluorescent dye to track the production of peroxide on the PEDOT pixel (Figure 1d). For the purpose of imaging, the size of the central PEDOT pixel was scaled down to 1 mm ø. The Amplex Red/horseradish peroxidase (HRP) mixture was added to the droplet prior to running the device under galvanostatic conditions with 1.9 µA (10 µA cm^−2^). The fluorescence signal was followed over the course of 10 min and imaged every minute. This yields a clear fluorescent signal which first originates over the pixel and diffuses outward. This experiment provides qualitative evidence that the central PEDOT cathode generates peroxide, which then travels outward over time.

To quantify the faradaic evolution of hydrogen peroxide, we employed two separate techniques which give different important information. The first is the HRP/tetramethylbenzidine (HRP/TMB) optical absorption assay^[^ ^45^ ^]^ (Figure 1e). The second is amperometric sensing with peroxide‐specific and oxygen‐specific microsensor probes (Figure 1f–j). The HRP/TMB assay is used to quantify the average concentration of H_2_O_2_ in the volume of electrolyte. This can therefore be used to compute the faradaic efficiency (FE) of peroxide production by using the total charge that is passed through the electrochemical circuit. For accurate measurement of FE, experiments are performed with the palladium electrode covered with a rubber block, and a platinum counter electrode placed in a separate anode compartment separated from the cathode chamber by an agarose salt bridge. This is critical to confine the produced H_2_O_2_ to the cathode chamber and prevent its diffusion and subsequent oxidation at the anode. This would otherwise yield an underestimated FE. Figure 1e shows the amount of peroxide produced over a 20‐min long galvanostatic (−1.9 µA = 10 µA cm^−2^) experiment, as well as the corresponding faradaic yield. As oxygen is consumed and [H_2_O_2_] grows, the faradaic yield is 76% ± 2% over 20 min. The remaining charge is consumed by two side processes, Equations (3) and (4): peroxide reduction and hydrogen evolution, respectively.

The HRP/TMB assay allows quantitative faradaic yield calculations, this method cannot probe if there are gradients in [H_2_O_2_] over the electrolyte volume. In reality, the concentration of peroxide should vary as a function of time and position relative to the cathode. It is logical that the concentration of H_2_O_2_ should be higher closer to the cathode, especially at the start of the experiment. For this reason, amperometric microsensors are very useful to probe the concentration in a position‐dependent way. We used two types of amperometric sensors: one for peroxide and the other for dissolved oxygen. We placed these sensors at fixed positions above the peroxide‐evolving cathode (Figure 1f), and ran galvanostatic experiments with the concentric PEDOT:PSS versus palladium design. Measuring at different distances reveals a clear gradient in evolved peroxide and consumed O_2_ (Figure 1g,i). Over the course of a 10‐min galvanostatic experiment with 1.9 µA of current, large differentials in [H_2_O_2_]/[O_2_] are apparent. At a distance 250 µm above the center of the cathode, peroxide concentration rises to 150 µm, while simultaneously the concentration of dissolved oxygen drops from an initial equilibrium value to 260 µm down to 140 µm. The ratio [H_2_O_2_]/[O_2_] is 0.57 at this position. Measuring at points further away from the cathode surface, the ratio [H_2_O_2_]/[O_2_] declines. At a height of 750 µm, the ratio is 0.22. We performed cyclic measurements with current set to 1.9 µA, 10 min on, followed by 10 min off (Figure 1h). These cycles show a reproducible peroxide concentration increase followed by decline when current is shut off. The corresponding oxygen consumption and recovery curves (Figure 1j) represent a mirror image to the peroxide measurements. Peroxide declines when current is off due to its disproportionation at the palladium electrode (Equation (7)), as well as the minor contribution of consumption by the amperometric sensor current (nA level currents). In this pixel configuration, the peroxide concentration has a half‐life of 2.3 min (Figure S2, Supporting Information). Oxygen concentrations recover when current is shut off, as [O_2_] is replenished by dissolution and diffusion of O_2_ from the air around the electrolyte drop. As expected, placing the peroxide sensor over the palladium electrode yields much lower peroxide values, by about an order of magnitude (Figure S3, Supporting Information). Meanwhile, oxygen concentrations measured above palladium increase above baseline values during oxidation of H_2_O_2_ to O_2_ (Figure S4, Supporting Information). The factors at play in the differential gradients of O_2_ and H_2_O_2_ are complex, as they will depend on many variables such as current, faradaic efficiency, diffusion coefficients, and temperature. The interplay of these factors is best evaluated using computational methods which will be addressed in the following section. From these experimental measurements, however, the picture that emerges is that peroxide and oxygen concentrations can be varied significantly, with the largest changes being localized at the PEDOT cathode. The palladium anode functions as a guard electrode, blocking peroxide diffusion and maintaining relatively constant levels of dissolved oxygen. PEDOT is therefore a good catalyst for accumulation of peroxide, in accordance with earlier reports.^[^ ^39^ ^]^ We performed also control experiments with bare titanium as a cathode, finding that equilibrium concentrations of peroxide are about an order of magnitude lower than those formed over PEDOT electrodes (Figure S5, Supporting Information).

### 2.2 Computational Modeling of Oxygen and Peroxide Gradients

**Figure 2:**
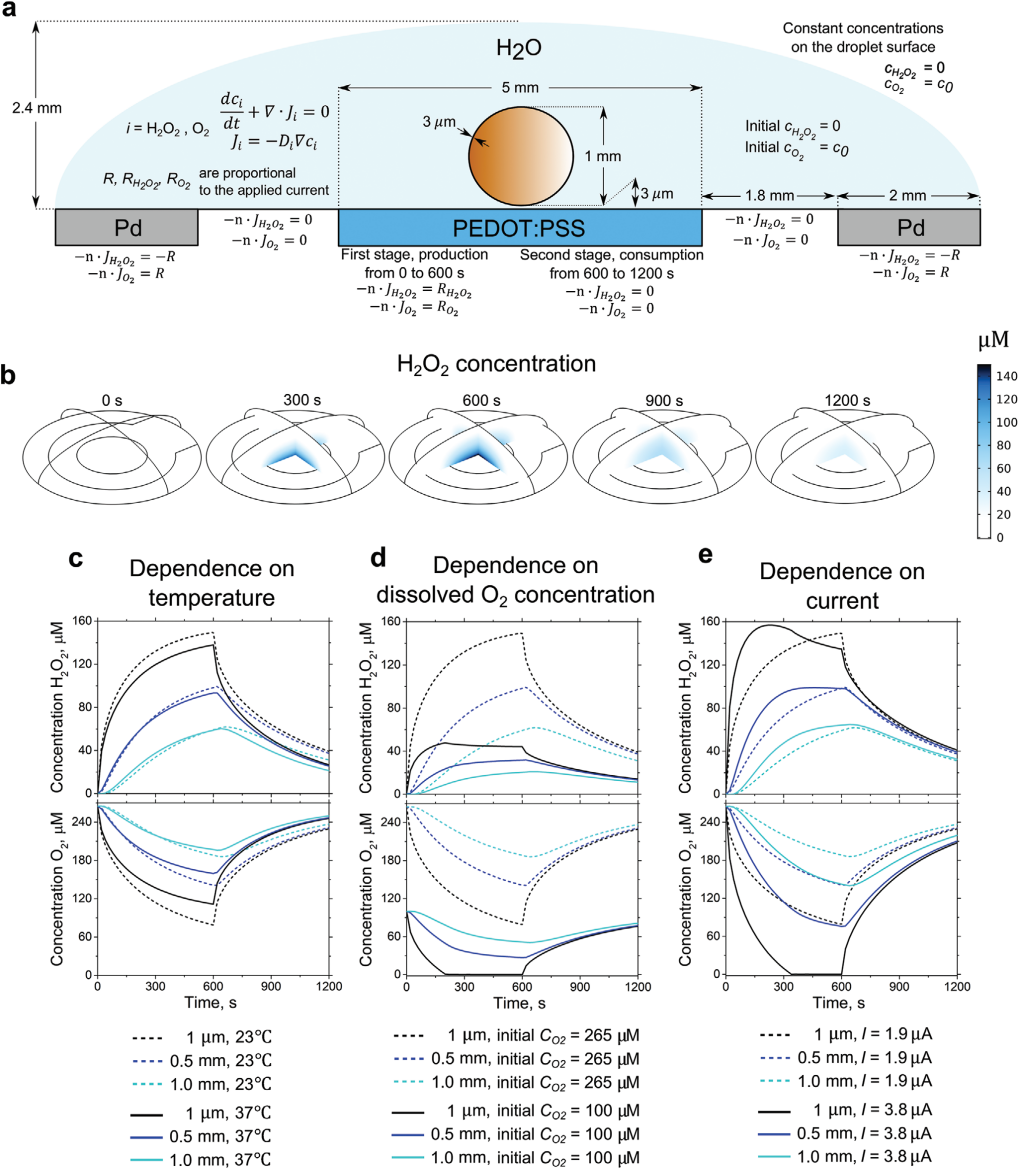
Finite element modeling illuminates the interplay of O_2_ and H_2_O_2_ gradients. a) 2D slice of the 3D model, used during the simulations of H_2_O_2_ and O_2_ diffusion with given boundary conditions and regions of production/consumption of diffused species. The geometry reproduces that of the electrophysiology experiments on oocytes (brown sphere in the center). b) 3D concentration profiles of H_2_O_2_ at different times from 0 to 1200 s (Current is turned on during the first 600 s, then switched off). c–e) Calculated [H_2_O_2_] (top row) and [O_2_] (bottom row) at a different height from the center of the PEDOT pixel: 1 µm, 0.5 mm, 1 mm. The calculations closely follow the microamperometry experimental setup. Each calculation shows the effect of a different critical parameter: c) temperature, d) initial O_2_ concentration, and e) applied current. At a distance of 1 µm, O_2_ concentration drops to values below the measurable threshold, indicated by the flattening of the black lines.

The relative concentrations of peroxide and O_2_ will depend on time, position, and the respective electrochemical and disproportionation reactions. The concentrations can be computed over time and position using diffusion coefficients of H_2_O_2_ and dissolved O_2_ and the geometry of the cathode, anode, and water droplet. Based on this, we constructed a finite element model, with parameters and boundary conditions given in **Figure** **2**a. We perform calculations on the “bare” device geometry, as well as considering the addition of the *X. laevis* oocyte that is used in the electrophysiology experiments in Section 2.3. Several values, such as the initial concentration of oxygen *c*_0_, production rate *R*, and temperature of the system *T*, are given. The initial equilibrium concentration of dissolved oxygen in the electrolyte solution was measured to be *c*_0_ = 265 µm. A production rate of H_2_O_2_ is dependent on the current applied to the PEDOT cathode, which in all experiments was −1.9 µA. The temperature of the solution was set to 23 °C. These parameters were used to reproduce the experimental setup in simulations. The simulation process was divided into two parts, namely the production stage that goes for 600 s and the consumption stage for the next 600 s when produced peroxide is freely diffusing over the solution droplet and can be decomposed at the palladium surface. 3D concentration profiles of H_2_O_2_ are depicted in Figure 2b and Video S1, Supporting Information. An increase of H_2_O_2_ concentration is observed on the time scale 600 s with a maximum value near the PEDOT surface and gradient decrease with the distance due to diffusion. During the next 600 s, no current is applied to PEDOT, and therefore no peroxide is produced. Over this time, the available H_2_O_2_ continues diffusing further into the droplet medium. Simultaneously, oxygen will diffuse down its gradient toward the PEDOT to compensate for its consumption. The net decrease in total peroxide concentration in the droplet occurs in two possible ways: consumption on the palladium electrode or evaporation from the droplet surface. To better understand the process, the influence of each of the critical parameters: T, c_0_, and R were studied individually and compared with the data obtained in experiment (Figure 2c–e). The temperature of the solution strongly influences the diffusion coefficients of the diluted species. Higher temperature means higher diffusion coefficients and faster spreading of the peroxide across the droplet. The change of the temperature from 23 to 37 °C leads to the increase of $D_{H_{2}O_{2}}$ and $D_{O_{2}}$ of ≈ 35%. At 37 °C, the recorded $c_{H_{2}O_{2}}$ at the end of the production stage (600 s) is almost the same, except the narrow µm region in the vicinity of the PEDOT surface. As for oxygen, the impact of increased temperature is more pronounced. Compensation of oxygen losses happens noticeably faster. (Figure 2c).

The initial content of O_2_ (*c*_0_) in the droplet plays a crucial role in peroxide production. O_2_ serves as a “fuel” for the reaction, and its supply at the reaction area (PEDOT surface) defines H_2_O_2_ flux. Reducing the *c*_0_ from 265 to 100 µm results in the decrease of H_2_O_2_ production roughly by three times (Figure 2d). While the production in the first seconds is still relatively high, from 200 s all of the dissolved oxygen near PEDOT becomes fully depleted. Under these conditions of O_2_ starvation, peroxide production will cease. When this happens under galvanostatic conditions, the current will be primarily consumed via the reduction of peroxide to water (Equation (2)).

A similar situation takes place when the peroxide production rate is increased instead of decreasing the oxygen content. Production of peroxide can be boosted by the increase of current applied to PEDOT. Assuming doubled current compared to the experimental conditions (3.8 µA), peroxide production significantly increased during the first 100 s simultaneously with the consumption of all available dissolved oxygen near PEDOT (Figure 2e). After the first 100 s, the faradaic efficiency of the process drops from 95% to 20%, and H_2_O_2_ production therefore drastically decreases. As a result, despite a rapid start, the resulting concentrations of peroxide after 600 s of production are almost the same for *I* = 1.9 µA or *I* = 3.8 µA. Simultaneously, the oocyte would suffer heavy oxygen starvation due to oxygen depletion at the PEDOT surface. Even at a distance of 1 mm from the pixel, $c_{O_{2}}$ is twice smaller than its initial value. It is important to note that due to the fact that both H_2_O_2_ and O_2_ are uncharged small molecules, they diffuse readily through cell membranes. When the oocyte is included in the model (as shown in Figure 2a), its effects on the H_2_O_2_ and O_2_ gradients are negligible (Figure S6, Supporting Information), that is, both molecules freely move through the oocyte as through electrolyte alone.

The obtained simulation indicates how peroxide production by a PEDOT pixel proceeds according to three interconnected factors: production rate, availability of dissolved oxygen, and diffusion coefficients of the diluted species. The formation of the peroxide on the surface of PEDOT is followed by its diffusion further into the droplet medium with an apparent gradient in concentration. Simultaneously, depletion of oxygen happens near the surface of the pixel, which invokes diffusion of the available dissolved oxygen from the droplet toward PEDOT. The calculated oxygen and peroxide concentrations over time closely match what is found experimentally (Figure 1g,h) using microamperometric probes. For the model to match experiment in terms of peroxide accumulation over cycles, the boundary condition at the edge of the droplet assumes peroxide concentration is zero outside the droplet (Figure S7, Supporting Information). This indicates that the model is in fact accurate and can be applied to understand how oxygen and peroxide concentrations can vary under different conditions and device geometries. If the goal is to maximize the production of H_2_O_2_, the supply of sufficient dissolved O_2_ is critical. In the case of a too large production rate, a rapid decrease of the faradaic efficiency occurs, accompanied by oxygen depletion. The effect of droplet volume loss due to evaporation can also be easily accounted for in the model. The results (Figure S8, Supporting Information) demonstrate that marked effects to oxygen and peroxide gradients are not present until evaporation loss is around 50% of the original droplet volume.

### 2.3 Electrochemical H_2_O_2_ Delivery Facilitates Opening of the Human Heteromeric Kv7.2/7.3 M‐Channel

Following characterization of H_2_O_2_ generation at the PEDOT/electrolyte interface, we set our attention to using these devices to modify electrophysiological properties of ROS‐sensitive voltage‐gated ion channels. For this purpose, we chose *X. laevis* oocytes to monitor the faradaic effects of H_2_O_2_ at a single cell level. *X. laevis* oocytes are a well‐established single‐cell electrophysiology model system, known for its robustness, large size (1 mm ø), and ability to reliably express a chosen type of ion channel.^[^ ^46^ ^]^ To test the PEDOT‐mediated H_2_O_2_ delivery we expressed human heteromeric Kv7.2/7.3 M‐channels in *X. laevis* oocytes. The sensitivity of Kv7.2, Kv7.4, and Kv7.5 channels to H_2_O_2_, and the lack thereof for Kv7.1 and Kv7.3 channels, has been established previously in a Chinese hamster ovary cell model conducted by Gamper et al.^[^ ^23^ ^]^ This paper reports that currents generated both by Kv7.2 and Kv7.2/7.3 are augmented by application of extracellular H_2_O_2_. The proposed mechanism behind the peroxide sensitivity is semi‐reversible oxidative modification of three cysteine residues in the intracellular S2‐S3 linker.

**Figure 3:**
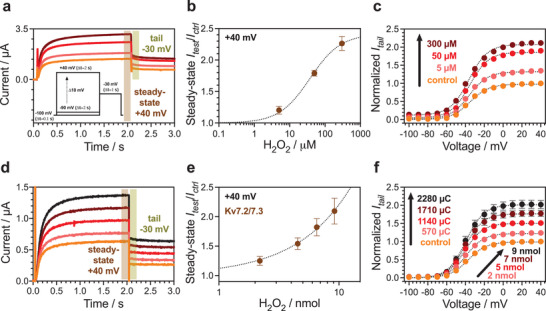
Faradaic delivery of H_2_O_2_ modifies currents in H_2_O_2_‐sensitive Kv7.2/7.3 ion channels. Panels a–c): Effect of H_2_O_2_ delivered via perfusion on the Kv7.2/7.3 M‐channel. a) Representative current traces for one oocyte before (control, light orange) and after perfusing H_2_O_2_ with concentrations of 5, 50, and 300 µm (indicated by darker color gradient, as in (c) at following test voltages +40 mV (steady‐state), −30 mV (tail). Inset: voltage protocol showing 3 s‐long test pulses applied throughout all TEVC experiments. b) Relative change in steady‐state current at +40 mV (*I*_ss,test_/*I*_ss,ctrl_, Table 1) for 5, 50, and 300 µm H_2_O_2_ (mean ± SEM, *n* = 3). Concentration‐response curve fitted using Equation (8); *A* = 2.41 ± 0.07, *C*_50_ = 37.39 ± 6.86 µm. c) Representative normalized *I*_tail_ for one oocyte, curve fitted with Equation (9). Panels d–f): Activation of Kv7.2/7.3 channels upon electrochemical H_2_O_2_ delivery with PEDOT faradaic pixel devices. d) Representative steady‐state (+40 mV) and tail current (−30 mV) current traces measured in a single oocyte placed on top of the PEDOT cathode before (control) and after exposure to 2.19, 4.53, 6.53, and 9.17 nmol H_2_O_2_ (indicated by darker color gradient, as in (f). e) Relative change in steady‐state current ratios at +40 mV (*I*_ss,test_/*I*_ss,ctrl_, Table 1) (mean ± SEM, *n* = 11). Concentration‐response curve fitted using Equation (8), although a saturation in the curve is not reached. f) Normalized *I*_tail_ plotted against preceding test voltage, data fitted with Equation (9) (mean ± SEM, *n* = 11, in total 10 different PEDOT faradaic pixel devices were tested).

All experiments on oocytes were conducted using the conventional two‐electrode voltage‐clamp technique (TEVC) in the arrangement as shown in Figure 2a (experimental setup photos shown in Figure S9, Supporting Information). In the voltage protocol used to measure K^+^ currents, the holding potential was set to −100 mV. Depolarizing test steps between −100 and + 40 mV (2 s, 10 mV increments) were used to open the channel, followed by a step back to −30 mV (1 s) to measure tail currents (**Figure** **3**a inset). Prior to testing the PEDOT pixel device, it was essential to confirm that the Kv7.2/7.3 channels expressed in *X. laevis* oocytes were sensitive to H_2_O_2_ (Figure 3a–c). An initial control recording before perfusion of H_2_O_2_ was performed to quantify the current generated under control conditions. This was followed by perfusion experiments in which different concentrations of H_2_O_2_ (5, 50, and 300 µm) were consecutively introduced into the bath chamber. After perfusing each concentration of H_2_O_2_, we performed TEVC recordings using the same voltage protocol as in the control experiment. Depolarizing the membrane potential resulted in an opening of the voltage‐gated Kv7.2/7.3 channels and subsequent outward flow of potassium ions, which was recorded as a positive current that reaches saturation (steady‐state) 2 s after onset of depolarizing voltage pulses above −30 mV. Figure 3a depicts representative current traces generated by stepping to a test voltage of +40 mV. A clear augmentation of the steady‐state current (*I*_ss_) at +40 mV was visible with increasing H_2_O_2_ concentrations, 300 µm H_2_O_2_ increased the current by 2.26 ± 0.06 ‐fold (Figure 3a,b, **Table** **1**). In the concentration‐response curve the maximum increase, given as the ratio *I*_ss_,_test_/*I*_ss_,_control_, is 2.41 ± 0.07, and the half maximal concentration 37.4 ± 6.9 µm (Figure 3b, Table 1). To determine if the H_2_O_2_‐induced augmentation at +40 mV was a result of an increase in the maximal conductance (*G*_MAX_) alone or accompanied by a shift in the voltage dependence of channel activation, we quantified the instantaneous tail current (when stepping to −30 mV after each test voltage, Figure 3a) under control conditions, and after perfusion with H_2_O_2_. Figure 3c shows representative tail currents (*I*_tail_) plotted versus the preceding test voltage, normalized to the control, clearly demonstrating the ability of H_2_O_2_ to increase the maximal conductance (*G*_MAX_) in a concentration‐dependent manner, while having no effect on the voltage dependence for channel activation (ΔV_50_, Figure 3c, Table 1). Taken together, this demonstrates that Kv7.2/7.3 channels expressed in *X. laevis* oocytes are susceptible to H_2_O_2_ delivered via perfusion, with H_2_O_2_ augmenting the current generated by Kv7.2/7.3 channels by increasing the conductance.

**Table 1:** Overview of numerical values for relative steady‐state current *I*_ss_, maximal conductance (*G*_MAX_), and voltage shift Δ*V*_50, test_ determined for constructs Kv7.2/7.3 WT (wild type) and Kv7.2/C150A/C151A/C152A under different configurations, including delivery of H_2_O_2_ via perfusion or the faradaic pixel device (here “Faradaic”). For perfusion, a concentration of peroxide is given (µm), for faradaic pixels, the molar amount (nmol) is indicated

Construct	Configuration	H_2_O_2_ amount (or time)	*I*_ss,test_/*I*_ss,ctrl_ [@+40 mV]	*G*_MAX,test_/ *G*_MAX,ctrl_	Δ*V*_50,test_ [mV]	*n*
Kv7.2/7.3 WT	Perfusion	5 µm	1.20 ± 0.04	1.21 ± 0.07	1.05 ± 0.85	3
Kv7.2/7.3 WT	Perfusion	50 µm	1.79 ± 0.03	1.86 ± 0.09	2.60 ± 1.14	3
Kv7.2/7.3 WT	Perfusion	300 µm	2.26 ± 0.06	2.38 ± 0.14	3.47 ± 2.16	3
Kv7.2/7.3 WT	Faradaic	2.19 nmol	1.25 ± 0.02	1.24 ± 0.03	−2.21 ± 0.61	11
Kv7.2/7.3 WT	Faradaic	4.53 nmol	1.54 ± 0.03	1.51 ± 0.05	−3.17 ± 0.65	11
Kv7.2/7.3 WT	Faradaic	6.53 nmol	1.82 ± 0.04	1.78 ± 0.08	−2.85 ± 0.69	11
Kv7.2/7.3 WT	Faradaic	9.17 nmol	2.10 ± 0.07	2.02 ± 0.11	−2.33 ± 0.71	11
Kv7.2/7.3 WT	Time‐match	10 min	1.10 ± 0.05	1.10 ± 0.03	−4.29 ± 1.31	3
Kv7.2/7.3 WT	Time‐match	20 min	1.15 ± 0.04	1.17 ± 0.04	−5.22 ± 1.55	3
Kv7.2/7.3 WT	Time‐match	30 min	1.19 ± 0.03	1.22 ± 0.05	−5.82 ± 1.92	3
Kv7.2/7.3 WT	Time‐match	40 min	1.23 ± 0.04	1.26 ± 0.02	−5.57 ± 1.29	3
						
Kv7.2/C150A/C151A/C152A	Perfusion	500 µm, 10 min	0.97 ± 0.09	1.08 ± 0.09	2.30 ± 0.76	3
Kv7.2/C150A/C151A/C152A	Perfusion	500 µm, 20 min	1.02 ± 0.11	1.17 ± 0.10	3.96 ± 1.13	3
Kv7.2/C150A/C151A/C152A	Faradaic	2.19 nmol	1.06 ± 0.04	1.09 ± 0.04	−0.63 ± 0.99	3
Kv7.2/C150A/C151A/C152A	Faradaic	4.53 nmol	1.22 ± 0.08	1.28 ± 0.09	−1.01 ± 0.65	3
Kv7.2/C150A/C151A/C152A	Faradaic	6.53 nmol	1.36 ± 0.11	1.46 ± 0.11	−1.72 ± 0.64	3
Kv7.2/C150A/C151A/C152A	Faradaic	9.17 nmol	1.41 ± 0.14	1.51 ± 0.15	−1.27 ± 0.75	3

The H_2_O_2_ effect on relative steady‐state current at +40 mV (*I*_ss_), maximal conductance (*G*_MAX_), and voltage dependence of channel activation (V_50_) was determined as described in Experimental Section. Data shown as mean ± SEM. *n* denotes the number of oocytes recorded in each experiment. Statistically significant effects (defined as *P* < 0.05) are highlighted in bold and determined using one‐sample *t*‐test to compare to a hypothetical value of 1 (for relative *I*_ss_ or *G*_MAX_) or 0 (for Δ*V*_50_).

After having established a reliable oocyte model, expressing H_2_O_2_‐sensitive Kv7.2/7.3 channels, we proceeded with probing the electrochemical delivery of H_2_O_2_ with the PEDOT faradaic pixel device. We measured oocytes using TEVC in the configuration as shown in Figure 2a, with the cell positioned in the center of the PEDOT cathode. TEVC recordings were carried out while simultaneously operating the faradaic pixel under galvanostatic conditions. Adapting the cycle protocol (on, off) introduced previously (Figure 1i), we ran four cycles with the PEDOT device and measured steady‐state and tail currents during each off‐period using the voltage protocol established in initial perfusion recordings (Figure S10). Contrary to the perfusion experiments, where the [H_2_O_2_] is assumed to be homogenous throughout the measurement solution, from the experiments discussed in Sections 2.1 and 2.2, we know that a gradient of [H_2_O_2_] will exist in the experiment. The bottom part of the oocyte that is closest to the PEDOT film surface will experience the highest amount of H_2_O_2_ while the top half is exposed to lower H_2_O_2_ amounts. Therefore, for all experiments with the faradaic pixel (Figure 3d–f), instead of concentration [H_2_O_2_], we refer to molar amount of H_2_O_2_ (nmol), and in terms of the electrical charge (µC) that has passed through the PEDOT during active operation. As in the prior perfusion experiments, we started measurements with a control recording of the oocyte without H_2_O_2_. Not only does a control measurement serve as point of reference but is also a good indicator for the quality of Kv7.2/7.3 channel expression and an assurance that the PEDOT device does not harm the oocyte cell membrane.

Figure 3d shows representative steady‐state and tail current traces recorded at +40 and −30 mV, respectively. A clear rise in *I*_ss_ at + 40 mV (Figure 3d,e, Table 1), and *G*_MAX_ (Figure 3f, Table 1), is apparent with increasing amount of H_2_O_2_ that is being delivered to the oocyte after each on cycle with the PEDOT device; 2.19, 4.53, 6.53 and 9.17 nmol of H_2_O_2_ for cycle 1–4, respectively (Figure 3d,e, Table 1). After the fourth cycle the steady‐state current increased by 2.10 ± 0.07‐fold, similar to what is expected with around 100 µm H_2_O_2_ in the perfusion experiments (Figure 3b). Since the oocytes become less stable over time (e.g., because of membrane leakage) we did not run more than 4 cycles with the PEDOT device (≈45 min), which explains why a saturation in the concentration‐response curve is not reached (Figure 3e). We also could see a small but statistically significant shift in the voltage dependence of activation (−2–3 mV), but independent of the number of PEDOT device cycles (Figure 3f, Table 1). The electrochemical H_2_O_2_ delivery via the PEDOT device thus clearly increases the maximal conductance of the Kv7.2/7.3 channel, similar to H_2_O_2_ in the perfusion experiments in the micromolar range.

**Figure 4:**
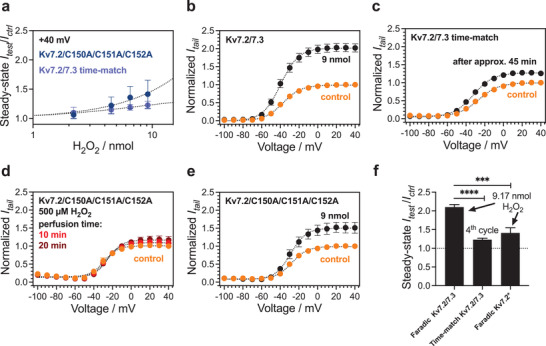
Increased potassium currents are primarily caused by delivered H_2_O_2_ acting on a cysteine triplet in Kv7.2. Panels (b–e) are fitted with sigmoidal Boltzmann function (Equation (9)). Panel a) concentration‐response plots for electrochemical H_2_O_2_ delivery, showing the results for steady‐state currents at +40 mV in Kv7.2/C150A/C151A/C152A and a time‐matched control with device switched off for Kv7.2/7.3 (mean ± SEM, *n* = 3 (Kv7.2/C150A/C151A/C152A), *n* = 3 (Kv7.2/7.3 time‐match)). Panels b,c) comparison between TEVC recordings of Kv7.2/7.3 after delivering 9.17 nmol of H_2_O_2_ via the PEDOT cathode (panel b, ± SEM, *n* = 11; includes same data as in panel 3(f) and during a 45 min‐long time‐match control without H_2_O_2_ where the cathode is not operated (panel c, mean ± SEM, *n* = 3). Panel d) TEVC recordings of H_2_O_2_‐insensitive Kv7.2/C150A/C151A/C152A after perfusing 500 µm H_2_O_2_ for 10 and 20 min (mean ± SEM, *n* = 3). Panel e) exposure of Kv7.2/C150A/C151A/C152A to 9.17 nmol H_2_O_2_ via PEDOT cathode (mean ± SEM, *n* = 3). Panel f) statistical analysis and comparison of relative steady‐state *I*_ss,test_/*I*_ss,ctrl_ obtained for Kv7.2/7.3 and Kv7.2/C150A/C151A/C152A upon delivery of 9.17 nmol H_2_O_2_ by the faradaic pixel device. Middle bar “Time‐match Kv7.2/7.3” shows relative steady‐state *I*_ss,test_/*I*_ss,ctrl_ of oocytes recorded without operating the PEDOT cathode in time‐match experiments, (mean ± SEM, *n* = 3–11, asterisk: *p* < 0.0001(****), *p* < 0.005 (***)).

In order to confirm that the Kv7.2/7.3 channel activation is in fact being caused by electrochemically‐produced H_2_O_2_, we conducted time‐match controls (**Figure** **4**). These controls are obtained by doing TEVC measurements of oocytes expressing Kv7.2/7.3 using the same cycle interval as before, but without operating the PEDOT device (Figure S10, Supporting Information). After 30–40 min (cycle 3–4), *I*_ss_ at + 40 mV and *G*_MAX_ increased marginally, ≈1.2–1.25‐fold (Figure 4a,c, Table 1), while no significant effect was seen for the voltage dependence of channel activation (ΔV_50_, Figure 4c, Table 1). Such a small rise in current amplitude is normal during longer oocyte recordings. The augmentation is significantly lower compared to when the PEDOT device is running (Figure 4f), thus suggesting the electrochemically produced H_2_O_2_ is responsible for effect.

As a further validation of the peroxide‐induced current enhancement, we also tested if the current increase for the Kv7.2/7.3 channel can be attributed to the oxidative modification of triple cysteine residues in the Kv7.2 subunit.^[^ ^23^ ^]^ Guided by experiments showing that a triplet of cysteines in the intracellular S2‐S3 linker is responsible for H_2_O_2_ effects in Kv7.4, we constructed a Kv7.2 mutant with the corresponding cysteines mutated to alanines (i.e., Kv7.2/C150A/C151A/C152A). The lack of the triple cysteine residues also made the Kv7.2 channel insensitive to peroxide, 500 µm H_2_O_2_ had no effect on *I*_ss_ at +40 mV, *G*_MAX_, or ΔV_50_ in the perfusion experiments, even after 20 min incubation time (Figure 4d, Table 1). When the faradaic pixel device was tested on the Kv7.2 mutant channel (same 4 cycles as before, Figure S10, Supporting Information) there was no significant increase in *I*_ss_ at + 40 mV or *G*_MAX_ (Figure 4a,e,f, Table 1). The voltage dependence of channel activation was also unchanged (Figure 4e, Table 1). This suggests that the faradaic pixel is acting upon the electrophysiology of the Kv7.2/7.3 channels primarily through oxidation of the triple cysteine residues in the Kv7.2 subunit by electrochemical delivery of H_2_O_2_.

Altogether, the oocyte experiments show that the faradaic pixel device can modify the behavior of human neuronal Kv7.2/7.3 channels via electrochemical dosing of peroxide, demonstrating the potential of the faradaic pixel device to control H_2_O_2_‐sensitive physiological responses.

## 3 Discussion and Conclusions

O_2_ reduction is the key reaction in cellular respiration. Respiration pathways also produce ROS as byproducts: peroxide, superoxide, and hydroxyl radicals. The balance of these species is critical to homeostasis, as high levels can result in toxic effects, while lower concentrations mediate important signaling pathways. Artificial manipulation of oxygen chemistry in biological systems using electrical devices could be a powerful tool for basic research and eventually biomedical devices. In this work, we report an electrochemical device fabricated using conventional microfabrication techniques which is capable of tuning O_2_/H_2_O_2_ levels in a localized way. Due to the manufacturing method, these devices can be obtained in any desired size or layout. For example, the faradaic pixel can be made in larger configurations to target tissues or organs, or alternatively shrunken down into microelectrode array formats appropriate for experiments with cultured neurons or brain slices. Regardless of size scale, the device acts as an H_2_O_2_ delivery electrode that consumes dissolved O_2_ as a “fuel.” The electrochemical circuit is completed by a counter electrode, which is made out of palladium in order to efficiently eliminate the peroxide which is produced. This design can be used to ensure the localization of peroxide delivery. Device size and shape effects can be anticipated using the finite element model we have herein introduced. To visualize how the concept scales, the peroxide gradient for cathodes of size 5–0.5 mm diameter is shown in Figure S11, Supporting Information. Shrinking down the size of the peroxide‐generating cathode leads to the same peroxide concentration at the cathode's surface, but a much sharper spatial gradient. However, the effect of oxygen depletion is much less pronounced for smaller cathodes. The device centers around the redox properties of organic electronic materials. Based on their unique electrocatalytic^[^ ^47^, ^48^ ^]^ and photocatalytic^[^ ^49^, ^50^, ^51^ ^]^ properties combined with relative stability and biocompatibility, organic semiconductors seem uniquely poised to provide tunable ROS production for biological applications. Several organic small‐molecules as well as polymers have shown the ability to photo(electro)catalytically reduce oxygen to peroxide and/or superoxide.^[^ ^52^, ^53^ ^]^ Published results for using PEDOT as an electrocatalyst for peroxide evolution demonstrated stable operation for at least 24 h of galvanostatic current.^[^ ^39^ ^]^ In line with this, we repeatedly tested the PEDOT‐based faradaic pixels and did not observe any degradation or drop in performance. The materials choices of PEDOT, with palladium and titanium, with parylene‐c as a passivation layer, represent a robust combination that can be expected to yield stable and reproducible performance.

To demonstrate the functionality of this device, we turned our attention to modulation of Kv channels responsible for M‐currents. These channels are recognized as therapeutic targets for regulating neuronal excitability.^[^ ^28^, ^32^ ^]^ We suggest that peroxide‐mediated enhancement of potassium M‐currents can be a possible therapeutic approach to this end, and exploration of faradaic devices in excitable cell models and in vivo is a future goal. In this present work, using single‐cell electrophysiology protocols, we found that the peroxide‐delivery pixel devices unambiguously enhanced M‐currents of peroxide‐sensitive Kv7.2/7.3 ion channels. In contrast to findings by Gamper et al., perfusion with H_2_O_2_ did not cause a shift in the voltage dependence of the Kv7.2/7.3 channel gating, which can be most likely ascribed to the variation in expression system, *X. laevis* oocyte versus Chinese hamster ovary cells. Oocytes are a remarkably robust single cell model which enabled us to obtain highly reproducible results with low standard error. An important difference between electrochemically‐delivered peroxide and peroxide added by perfusion is that electrochemical generation involves the formation of a gradient of concentration. The concentration is higher at the electrode/oocyte interface and will be considerably less at the upper regions of the oocyte. This kind of gradient effect is analogous to our previous studies with extracellular stimulation electrodes^[^ ^54^, ^55^ ^]^ acting on *X. laevis* oocytes, where membrane depolarization is strongest at the electrode/oocyte interface.

The concepts we have introduced can be translated to other in vitro, as well as in vivo experimental conditions. Many experimental protocols are possible since the devices can be fabricated on essentially arbitrary substrates, including flexible plastics. To enable less‐invasive deployment of H_2_O_2_ delivery in electrophysiological experiments, it will be possible to drive such devices using photovoltaics. The concept of photofaradaic pixels was introduced by our group recently for the H_2_O_2_/glucose redox pair,^[^ ^56^ ^]^ and this can be adapted to the protocols discussed in this paper. We believe the results we have presented should stimulate research into reactive‐oxygen species mediated neuromodulation. In parallel, the findings should also encourage materials scientists and electrochemists to tap into the vast body of knowledge with oxygen redox chemistry to develop high‐performance devices for the manipulation of oxygen in physiological conditions.

## 4 Experimental Section

### Materials and Device Fabrication

All chemicals were purchased from Sigma Aldrich unless otherwise noted. Device samples were fabricated on glass microscope slides 3 × 1.” Following stepwise sonication in acetone (10 min), isopropanol (10 min), 2% Hellmanex III detergent (10 min), and deionized water (DI) (30 min) the glass slides were dried under N_2_ and treated with O_2_ plasma (0.6 mbar, 200 W) for ≈18 min (Diener electronic GmbH). Afterward, the slides were immediately coated with a 140 nm layer of titanium (Ti) in a thermal PVD system (base pressure 8 × 10^−7 ^Torr) at a rate of 2–5 Å s^−1^. The Ti back and Pd counter electrodes were patterned via photolithography. First, S1818 positive photoresist was spincoated (2000 rpm, 30 s) onto Ti and baked at 110 °C for 60 s. Using a Karl Süss MA6/BA6 mask aligner, the S1818 layer was exposed and subsequently developed in Microposit MF319. After development the S1818 layer was washed with DI water, dried under N_2,_ and baked again for 60 s at 110 °C. The exposed Ti areas without S1818 on top were removed via wet etching with NH_3_:H_2_O_2_:DI (1:2:1) solution. After successful etching, the Ti layer protected by S1818 was revealed by removing S1818 with acetone. The Pd counter electrode was patterned via metal lift‐off with acetone. Here, a S1818 photoresist layer served as mask. The glass area reserved for Pd was treated with O_2_ plasma (0.6 mbar) at 50 W for ≈18 min and first coated with 40 nm Ti followed by 30 nm Pd. Ti functioned as sticking layer between the glass substrate and Pd. After removal of S1818 the Ti and Pd electrodes were rinsed with DI, dried, and treated with O_2_ plasma (0.6 mbar) for ≈18 min at 200 W. In the next step, the electrodes were encapsulated with 2.2 µm of parylene C (Par‐C) via chemical vapor deposition (CVD, Diener electronic GmbH). During the CVD deposition, methacryloxypropyltrimethoxysilane (A‐174) was introduced into the vacuum chamber to increase the adhesion of the Par‐C layer. The Par‐C layer was patterned via photolithography with positive photoresist AZ 10XT (baked at 110 °C for 5 min, exposure time: 30 s) and removed above the Pd counter electrode and Ti/Pd contacts using reactive ion etching (RIE, O_2_, 200 W, 100 sccm, 700–800 s). The patterned Par‐C encapsulation layer was spin coated with micro‐90 (2%), an anti‐adhesive, and coated with a second Par‐C layer (CVD, 2.2 µm). In a final photolithography step the Par‐C layers above the central Ti back electrode were patterned with AZ 10XT positive photoresist and RIE (O_2_, 200 W, 100 sccm, 850 s). After removal of the AZ 10XT photoresist with acetone, the Ti film surface was treated with O_2_ plasma (50 W, ≈18 min, 0.6 mbar, Diener electronic GmbH) and immediately spin coated with fresh (3‐glycidyloxypropyl)trimethoxysilane (GOPS, stored under inert atmosphere) followed by a freshly prepared PEDOT:PSS solution composed of 0.25% 4‐dodecylbenzenesulfonic acid (DBSA), 5% dimethyl sulfoxide (DMSO) and 2% GOPS. The PEDOT:PSS formulation was prepared by sonicating PEDOT:PSS dispersion (PH 1000) for 10 min before adding DBSA. The mixture was stirred for 10 min until dissolution of DBSA. Following addition of GOPS the mixture was stirred for additional 5 min before spin coating (5000 rpm, 1000 acc, 30 s). Thereafter, the samples were annealed at 120 °C for 30 min and the top Par‐C layer was peeled off.

### Electrochemical Measurements

All cyclic voltammetry experiments were conducted with an Ivium technologies Vertex One potentiostat in a three‐electrode configuration composed of a platinum mesh counter electrode and an Ag/AgCl reference electrode (wire in 3 m KCl). The reference electrode was inserted into the droplet which was further connected to the counter electrode via a freshly prepared agarose salt bridge. The electrolyte used throughout all experiments in this paper, including electrophysiology measurements, was the “1K” extracellular medium composed of 88 mm NaCl, 1 mm KCl, 15 mm HEPES, 0.4 mm CaCl_2_, and 0.8 mm MgCl_2_ (pH 7.4, NaOH) solution prepared in DI water.

### Fluorescence Imaging of H2O2 Generation with Amplex UltraRed Reagent

A 10 mm stock solution was prepared by dissolving the content of one vial containing 1 mg of Amplex UltraRed reagent (Thermo Fisher Scientific) in 340 µL DMSO. The vial was stored in the dark at −20 °C. For the imaging experiment a working solution was obtained by mixing 50 µL of the 10 mm Amplex UltraRed stock solution with 100 µL HRP in 4.85 mL phosphate buffer solution (0.1 mol L^−1^, pH 7). The working solution was kept in the dark during the experiment. The imaging was conducted with an Eclipse Ni‐E Nikon fluorescence microscope in a darkroom using a TRITC filter. Prior to operation, the PEDOT:PSS pixel was covered with a 200 µL droplet of 1K electrolyte solution and 10 µL of the Amplex UltraRed/HRP mixture added to the droplet.

### HRP‐TMB UV–Vis Assay

The average H_2_O_2_ concentration generated above the central PEDOT:PSS pixel was determined spectrophotometrically via an HRP‐TMB assay with a Synergy H1 Microplate reader (BioTek Instruments, Inc.). The HRP‐TMB solution used for the assay was freshly prepared by mixing 2 µL HRP (solution 0.75 ng mL^−1^) and 5 µL 3,3′,5,5′‐tetramethylbenzidine (TMB, 10 mg mL^−1^ solution) in 993 µL disodium phosphate (Na_2_HPO_4_) (0.2 m)/citric acid (0.1 m) buffer solution (diluted 1:4 with DI water and filtered, pH 5.6). The 5 mm wide circular PEDOT:PSS/Ti film was operated galvanostatically at −1.9 µA by placing a 50 µL droplet of 1K electrolyte solution on top of the film surface. The droplet was connected to a platinum mesh counter electrode via a freshly prepared agarose salt bridge. After operating the PEDOT:PSS device for 5, 10, 15, and 20 min, a 35 µL aliquot was taken out of the 50 µL droplet and added to 265 µL HRP‐TMB assay solution and mixed thoroughly in a 96 well‐microplate. The colorimetric change of the solution upon the HRP‐catalyzed oxidation of TMB by H_2_O_2_ was measured at 653 nm. In order to quantify the H_2_O_2_ concentration a 5‐point calibration curve was established by measuring the colorimetric changes in 5 mixtures of H_2_O_2_ (1 mm stock solution in 1K) with HRP‐TMB assay solution with known H_2_O_2_ concentrations: 0, 10, 20, 30, and 40 µm.

### Amperometric Sensing of Peroxide and Oxygen

The quantification of H_2_O_2_ and O_2_ concentrations locally at the 1K electrolyte/PEDOT:PSS interface was performed with a 4‐Channel Free Radical Analyzer (World Precision Instruments, WPI) equipped with 2 mm wide H_2_O_2_ (ISO‐HPO‐2, WPI) and O_2_ (ISO‐OXY‐2, WPI) sensors (polarization voltages: *V*_H2O2_ = +450 mV & *V*_O2_ = +700 mV). Lab‐Trax4/16 (WPI) and LabScribe software (version 4.31) were used for recording the sensor current. O_2_ and H_2_O_2_ concentrations were determined separately. The sensors were mounted onto a stereotaxic frame which allowed accurate positioning of the sensor tip ≈250, 500, and 750 µm above the PEDOT:PSS film surface. A 5‐point calibration was established for the H_2_O_2_ sensor in 15 mL phosphate‐buffered saline (PBS) solution. The sensor tip was completely inserted into the PBS bath and the solution was continuously stirred. 165, 330, 750, and 750 µL of a 1 mm H_2_O_2_ stock solution in DI water were sequentially added to the PBS bath and changes in the sensor current were recorded continuously. A 2‐point calibration (0% and air‐saturated) was used to quantify the O_2_ concentration. Here, the O_2_ sensor was inserted into a commercially available calibration bottle (WPI) filled with DI water that was continuously stirred. First, the sensor current in air‐saturated DI water was measured before the content of the bottle was purged with N_2_. 1K solutions used throughout all measurements in this study were adjusted to room temperature (≈23 °C) over the course of several days. Contrary to HRP‐TMB experiments, the whole device architecture including the palladium counter electrode was covered with 200 µL 1K solution during all amperometric recordings.

### Electrophysiology Measurements with *X. laevis* Oocytes

*X. leavis* oocytes were either obtained via surgery and prepared for injections at Linköping University (approved by the Linköping Animal Care and Use Committee (Permit #1941)) or purchased from Ecocyte Bioscience. The *X. leavis* oocytes were injected 3–5 days prior to recording with 50 nL RNA containing 2.5 ng Kv7.2 and 2.5 ng Kv7.3 cRNAs (Kv7.2: GenBank accession no. NM_004518, Kv7.3: GenBank accession no. NM_004519, 1:1 molar ratio) and incubated at 8 °C. To ensure sufficient ion channel expression, the injected oocytes were stored at 16 °C overnight 1 day before experiments and used for recordings that were performed at room temperature. During perfusion experiments a pump (model ISM597D; Labinett Lab AB) was used, with a perfusion rate of 0.5 mL min^−1^. The Kv7.2/C150A/C151A/C152A mutant was constructed using site‐directed mutagenesis (QuickChange II XL with 10 XL Gold cells, Agilent) with correct sequence alteration ensured by sequencing at the Core Facility at Linköping University. *X. laevis* oocytes were injected 3–5 days prior to recording with 25 ng Kv7.2/C150A/C151A/C152A and incubated at 8 °C. All two‐electrode voltage clamp recordings (TEVC) were conducted with a whole‐cell amplifier (CA‐1B Dagan Corporation). 1K electrolyte was used throughout all measurements as extracellular medium. During measurements with the PEDOT:PSS faradaic pixel device both the Pd counter electrode as well as the PEDOT:PSS film were covered with a 200 µL droplet of 1K solution. The droplet was connected to both TEVC reference electrodes via a freshly prepared agarose salt bridge.

In the voltage protocol, the holding voltage was set to −100 mV. A multi‐step voltage protocol with test voltages ranging from −100 to +40 mV (10 mV increments, 2 s duration) was used to generate steady‐state currents, followed by a subsequent test voltage at −30 mV to generate tail currents (1 s duration). The sweep‐to‐sweep interval was 15 s, and the whole protocol took ≈3.5 min. Data were sampled at 5 kHz and filtered at 500 Hz.

After control recordings, the PEDOT:PSS faradaic pixel device was turned on in 4 on/off cycles (5 min ON, 5.5 min OFF, Figure S10, Supporting Information). To assess the effect on Kv7 channel opening, TEVC recordings were made after each on cycle. The whole protocol (control recording, plus 4 cycles) took ≈45 min. For the time‐match control experiments, that were conducted without operating the faradaic pixel, steady‐state and tail currents were recorded approximately every 10 min using the voltage protocol shown in Figure S10, Supporting Information. Normalized tail currents for the time‐matched control are shown in Figure 4c after ≈45 min. All electrophysiological data were analyzed with Clampfit 11.1.0.23, Matlab R2019a, and GraphPad Prism 9.

### Analysis of Currents

Potassium currents were leak‐subtracted and steady‐state currents (*I*_ss_) at +40 mV were quantified at the end of the voltage sweep. The relative change was determined as the ratio *I*_ss,test_/*I*_ss,ctrl_, and the concentration‐response curve was plotted using the following equation:

(8) $$\frac{I_{\mathrm{ss},\mathrm{test}}}{I_{\mathrm{ss},\mathrm{ctrl}}}=\frac{A}{1+\frac{C_{50}}{C}}\;$$

where *I*_ss,test_/*I*_ss,ctrl_ is the relative change in the steady‐state current, A the amplitude of the curve, C_50_ the concentration at which half‐maximal response occurs, and C the concentration (in µm or nmol).

Instantaneous tail current (*I*_tail_) was measured (after onset of the −30 mV step) and plotted against the preceding test voltage. Data were normalized to the control by dividing the data set by the current value of the control at +40 mV. To generate the conductance versus voltage (G(V)) curve in Figures 3, 4 the following Boltzmann function was fitted to the normalized *I*_tail_ data:

(9) $$G\;\left(V\right)=G_{\min}\;+\left(G_{\mathrm{MAX}}-G_{\mathrm{MIN}}\right)/\left\{1+\exp\left[\frac{\left(V_{50}-V\right)}{s}\right]\right\}\;$$

where *G*_MIN_ is the normalized minimal conductance, *G*_MAX_ the normalized maximal conductance, V_50_ the midpoint (i.e., the voltage needed to reach half the maximal conductance determined from the fit), V the test voltage, and s the slope of the curve (shared between test and control curve).

The relative change in *G*_MAX_ was calculated as the ratio *G*_MAX,test_/*G*_MAX,ctrl_, and the shift in the voltage dependence of activation (ΔV_50_) was calculated as *V*_50_,_test_ − *V*_50_,_ctrl_ from the Boltzmann fit. Oocyte data are summarized in Table 1.

### Statistical Analysis of Electrophysiological Data

Mean values presented in Section 2.3 are expressed as mean ± SEM if not stated otherwise. A one‐sample *t*‐test was used to compare to a hypothetical value of 1 (for relative *I*_ss_ or *G*_MAX_) or 0 (for ΔV_50_). When comparing groups in Figure 4f, one‐way ANOVA with Dunnett's multiple comparison test was used. Effects were considered statistically significant if *P* < 0.05.

### Computational Methods

Simulations were conducted using the finite element method, implemented in the COMSOL software package, version 5.5 (https://www.comsol.com/product‐download).

Schematic 2D slice of the modeled system can be found in Figure 2a, and the full 3D shape is in Figure 2b. The geometry of the simulation model was chosen to replicate experimental setup and consisted of three main regions: PEDOT pixel and palladium circle, covered with a water droplet. The parameters and sizes of model regions are depicted in Figure 2a.

There were two variables in the system: concentration of dissolved oxygen $c_{O_{2}}$ and concentration of peroxide $c_{H_{2}O_{2}}$. At a starting point, there was no H_2_O_2_ in the system, while the initial value of $c_{O_{2}}=c_{0}$, where *c*_0_ =  265 *μ*m was an equilibrium concentration of dissolved oxygen measured experimentally. The following equations express the diffusion of O_2_ and H_2_O_2_

(10) $$\frac{dc_{i}}{dt}+\nabla \cdot J_{i}=0$$

(11) $$J_{i}=\;-D_{i}\nabla c_{i}$$

where *i* goes for O_2_ and H_2_O_2_, *D_i_* are diffusivity coefficients, *J_i_* are fluxes of H_2_O_2_ and O_2_ molecules. Diffusion coefficients^[^ ^57^, ^58^ ^]^ at 23 °C are: $D_{H_{2}O_{2}}$= 1.8e‐9 s m^−2^, $D_{O_{2}}$= 2.5e‐9 s m^−2^.

Oocyte was simulated as a sphere covered with cell membrane. The distance between the oocyte and the PEDOT pixel was 3 µm. The oocyte cell membrane has diameter 3 µm. Diffusion coefficients of peroxide and oxygen molecule inside the oocyte were the same as in H_2_O. As for oocyte membrane, it has a slightly denser structure compared to water, and the corresponded diffusion coefficients of O_2_ and H_2_O_2_ were five times smaller.^[^ ^58^ ^]^ However, due to the small thickness of the membrane and oocyte envelope, it does not cause a significant impact on the results (Figure S6, Supporting Information). Overall, the diffusion process in oocyte domain was modeled with the same equations as in water, with the exception of increased diffusion coefficients in cell membrane.

Production and consumption of peroxide and oxygen were governed by the current dependent reaction rates *R*_i_ and were implemented through the boundary conditions (positive or negative fluxes of H_2_O_2_ and O_2_), specified in Figure 2a for each of the model regions (PEDOT:PSS, Pd, water). For each consumed oxygen, one peroxide molecule was produced on the PEDOT pixel. The reverse reaction leads to producing one O_2_ molecule for each H_2_O_2_ molecules′ consumption on the Pd surface. The following equations define reaction rates *R*_i_

(12) R=FI/2A

(13) $$f_{\mathrm{eff}}=\;0.2+0.8\frac{C_{O_{2}}^{\text{near}\;\text{PEDOT}}}{C_{O_{2}}^{\mathrm{saturated}}}$$

(14) $$R_{H_{2}O_{2}}=\;R\times f_{\mathrm{eff}}$$

(15) $$R_{O_{2}}=\;-R\times \left(f_{\mathrm{eff}}+\frac{1-f_{\mathrm{eff}}}{2}\right)$$

where *F* is the faraday constant, *I* is current applied to PEDOT, *A* is a pixel area, and *f*_eff_ is faradaic efficiency of peroxide production. Faradaic efficiency (Equation (13)) was designed to mimic H_2_O_2_ concentration plateau at the experimental results, caused by the lack of dissolved oxygen on the distance of 1 µm from pixel $C_{O_{2}}^{\text{near}\;\text{PEDOT}}$. $C_{O_{2}}^{\mathrm{saturated}}$ = 300 µm was the concentration of O_2_ required for maximal faradic efficiency. Reactions on the palladium electrode were reversed and lead to the decomposition of peroxide. If H_2_O_2_ molecules reach the Pd regions, they will be converted into dissolved oxygen with unity efficiency.

Switching between the production and consumption of molecules was implemented with the “events” interface and happened in two stages. Each of stages has its own boundary conditions on PEDOT:PSS surface (Figure 2a). The first stage happens when current was applied to PEDOT pixel (from 0 s to 600 s). During this stage, peroxide was produced and oxygen was consumed near the PEDOT surface simultaneously with the reversed reaction on palladium surface. The second stage happens when the current on the pixel was switched off (no inward or outward fluxes on PEDOT:PSS surface) with the reversed reaction on Pd still taking place (from 600 s to 1200 s). All boundary conditions, used during the implementation are defined in Figure 2a. The three simulation probe sites for detection of $c_{H_{2}O_{2}}$ and $c_{O_{2}}$ were located on top of the PEDOT pixel center at the distance of 1 µm, 0.5 mm, and 1.0 mm, covering all height of an oocyte starting from its lowest point.

## Conflict of Interest

The authors declare no conflict of interest.

## Author Contributions

S.I.L. and E.D.G. conceived the project idea. O.S.A. performed all device fabrication, electrochemical measurements, and assays, and most electrophysiological recordings. S.I.L. conducted original peroxide perfusion recordings. I.S. performed the calculations with finite‐element models. M.S.E. and M.J. contributed to sample preparation, experimental methods and design, and data analysis. The project was led and supervised by I.Z., S.I.L., and E.D.G. The manuscript was written and figures prepared with input from all coauthors.

## Supporting information

Supporting Information

Supplemental Video 1

## Data Availability

The data that support the findings of this study are available from the corresponding author upon reasonable request.

## References

[1] L. Y. Lee , J. Loscalzo , Systems Biology and Network Medicine: An Integrated Approach to Redox Biology and Pathobiology, Elsevier, New York 2020.

[2] H. J. Forman , F. Ursini , M. Maiorino , J. Mol. Cell. Cardiol. 2014, 73, 2. 24512843 10.1016/j.yjmcc.2014.01.018 PMC4048798

[3] Y. J. Suzuki , H. J. Forman , A. Sevanian , Free Radicals Biol. Med. 1997, 22, 269. 10.1016/s0891-5849(96)00275-4 8958153

[4] S. Di Meo , T. T. Reed , P. Venditti , V. M. Victor , Oxid. Med. Cell. Longevity 2016, 2016, 1245049. 10.1155/2016/1245049 PMC4960346 27478531

[5] K. M. Holmström , T. Finkel , Nat. Rev. Mol. Cell Biol. 2014, 15, 411. 24854789 10.1038/nrm3801

[6] R. Patel , L. Rinker , J. Peng , W. M. Chilian , Reactive Oxygen Species (ROS) in Living Cells (Eds: C. Filip , E. Albu ), IntechOpen, London 2018.

[7] B. D'Autréaux , M. B. Toledano , Nat. Rev. Mol. Cell Biol. 2007, 8, 813. 17848967 10.1038/nrm2256

[8] J. Roy , J. M. Galano , T. Durand , J. Y. Le Guennec , J. C. Y. Lee , FASEB J. 2017, 31, 3729. 28592639 10.1096/fj.201700170R

[9] H. Sies , C. Berndt , D. P. Jones , Annu. Rev. Biochem. 2017, 86, 715. 28441057 10.1146/annurev-biochem-061516-045037

[10] N. Di Marzo , E. Chisci , R. Giovannoni , Cells 2018, 7, 156. 10.3390/cells7100156 PMC6211135 30287799

[11] H. Sies , B. Chance , FEBS Lett. 1970, 11, 172. 11945479 10.1016/0014-5793(70)80521-x

[12] H. Sies , Curr. Opin. Toxicol. 2018, 7, 122.

[13] C. C. Winterbourn , Antioxid. Redox Signaling 2018, 29, 541. 10.1089/ars.2017.7425 29113458

[14] H. J. Forman , M. Maiorino , F. Ursini , Biochemistry 2010, 49, 835. 20050630 10.1021/bi9020378 PMC4226395

[15] G. P. Bienert , J. K. Schjoerring , T. P. Jahn , Biochim. Biophys. Acta, Biomembr. 2006, 1758, 994. 10.1016/j.bbamem.2006.02.015 16566894

[16] S. G. Rhee , Exp. Mol. Med. 1999, 31, 53. 10410302 10.1038/emm.1999.9

[17] C. Rampon , M. Volovitch , A. Joliot , S. Vriz , Antioxidants 2018, 7, 159. 10.3390/antiox7110159 PMC6262372 30404180

[18] H. Sies , Redox Biol. 2017, 11, 613. 28110218 10.1016/j.redox.2016.12.035 PMC5256672

[19] M. Genestra , Cell. Signalling 2007, 19, 1807. 17570640 10.1016/j.cellsig.2007.04.009

[20] H. Sies , J. Biol. Chem. 2014, 289, 8735. 24515117 10.1074/jbc.R113.544635 PMC3979367

[21] C. Glorieux , P. B. Calderon , Biol. Chem. 2017, 398, 1095. 28384098 10.1515/hsz-2017-0131

[22] N. Gamper , L. Ooi , Antioxid. Redox Signaling 2015, 22, 486. 10.1089/ars.2014.5884 PMC4323017 24735331

[23] N. Gamper , O. Zaika , Y. Li , P. Martin , C. C. Hernandez , M. R. Perez , A. Y. C. Wang , D. B. Jaffe , M. S. Shapiro , EMBO J. 2006, 25, 4996. 17024175 10.1038/sj.emboj.7601374 PMC1618113

[24] H. S. Wang , Z. Pan , W. Shi , B. S. Brown , R. S. Wymore , I. S. Cohen , J. E. Dixon , D. McKinnon , Science 1998, 282, 1890. 9836639 10.1126/science.282.5395.1890

[25] E. C. Cooper , K. D. Aldape , A. Abosch , N. M. Barbaro , M. S. Berger , W. S. Peacock , Y. N. Jan , L. Y. Jan , Proc. Natl. Acad. Sci. USA 2000, 97, 4914. 10781098 10.1073/pnas.090092797 PMC18332

[26] V. Barrese , J. B. Stott , I. A. Greenwood , Annu. Rev. Pharmacol. Toxicol. 2018, 58, 625. 28992433 10.1146/annurev-pharmtox-010617-052912

[27] N. Sahoo , T. Hoshi , S. H. Heinemann , Antioxid. Redox Signaling 2014, 21, 933. 10.1089/ars.2013.5614 PMC4116129 24040918

[28] F. Miceli , M. V. Soldovieri , P. Ambrosino , L. Manocchio , I. Mosca , M. Taglialatela , Curr. Med. Chem. 2017, 25, 2637. 10.2174/0929867324666171012122852 29022505

[29] D. L. Greene , N. Hoshi , Cell. Mol. Life Sci. 2017, 74, 495. 27645822 10.1007/s00018-016-2359-y PMC5243414

[30] P. Nappi , F. Miceli , M. V. Soldovieri , P. Ambrosino , V. Barrese , M. Taglialatela , Pfluegers Arch. 2020, 472, 881. 32506321 10.1007/s00424-020-02404-2

[31] A. Abd‐Elsayed , M. Jackson , S. L. Gu , K. Fiala , J. Gu , Mol. Pain 2019, 15, 174480691986425. 10.1177/1744806919864256 PMC6659175 31342847

[32] I. Rivera‐Arconada , C. Roza , J. A. Lopez‐Garcia , Front. Mol. Neurosci. 2009, 2, 10. 19680469 10.3389/neuro.02.010.2009 PMC2726036

[33] E. Esin , S. Yalcin , OncoTargets Ther. 2014, 7, 599. 10.2147/OTT.S60995 PMC4000251 24790459

[34] A. W. Phillips , R. Parameswaran , E. Lichter , J. Jeong , L. Meng , M. Burke , K. Koehler , Y. V. Lee , B. Tian , ACS Appl. Mater. Interfaces 2021, 13, 15490. 33779140 10.1021/acsami.0c23164

[35] I. Abdel Aziz , M. Malferrari , F. Roggiani , G. Tullii , S. Rapino , M. R. Antognazza , iScience 2020, 23, 101091. 32438318 10.1016/j.isci.2020.101091 PMC7240120

[36] M. R. Antognazza , I. A. Aziz , F. Lodola , Oxid. Med. Cell. Longevity 2019, 2019, 2867516. 10.1155/2019/2867516 PMC6462332 31049131

[37] F. Lodola , V. Rosti , G. Tullii , A. Desii , L. Tapella , P. Catarsi , D. Lim , F. Moccia , M. R. Antognazza , Sci. Adv. 2019, 5, eaav4620. 31598549 10.1126/sciadv.aav4620 PMC6764832

[38] J. Park , K. Jin , A. Sahasrabudhe , P. Chiang , J. H. Maalouf , F. Koehler , D. Rosenfeld , S. Rao , T. Tanaka , T. Khudiyev , Z. J. Schiffer , Y. Fink , O. Yizhar , K. Manthiram , P. Anikeeva , Nat. Nanotechnol. 2020, 15, 690. 32601446 10.1038/s41565-020-0701-x PMC7415650

[39] E. Mitraka , M. Gryszel , M. Vagin , M. J. Jafari , A. Singh , M. Warczak , M. Mitrakas , M. Berggren , T. Ederth , I. Zozoulenko , X. Crispin , E. D. Głowacki , Adv. Sustainable Syst. 2019, 3, 1800110.

[40] R. Valiollahi , M. Vagin , V. Gueskine , A. Singh , S. A. Grigoriev , A. S. Pushkarev , I. V. Pushkareva , M. Fahlman , X. Liu , Z. Khan , M. Berggren , I. Zozoulenko , X. Crispin , Sustainable Energy Fuels 2019, 3, 3387.

[41] W. Plieth , Electrochemistry for Materials Science, Elsevier, Amsterdam 2008.

[42] M. Grdeń , M. Łukaszewski , G. Jerkiewicz , A. Czerwiński , Electrochim. Acta 2008, 53, 7583.

[43] A. Plauck , E. E. Stangland , J. A. Dumesic , M. Mavrikakis , Proc. Natl. Acad. Sci. USA 2016, 113, E1973. 27006504 10.1073/pnas.1602172113 PMC4833276

[44] V. Gueskine , A. Singh , M. Vagin , X. Crispin , I. Zozoulenko , J. Phys. Chem. C 2020, 124, 13263.

[45] P. D. Josephy , T. Eling , R. P. Mason , J. Biol. Chem. 1982, 257, 3669. 6277943

[46] B. Hille , Ion Channels of Excitable Membranes, Sinauer Associates Inc, Sunderland, MA 2001.

[47] M. Warczak , M. Gryszel , M. Jakešová , V. Đerek , E. D. Głowacki , Chem. Commun. 2018, 54, 1960. 10.1039/c7cc08471d 29323369

[48] H. Rabl , D. Wielend , S. Tekoglu , H. Seelajaroen , H. Neugebauer , N. Heitzmann , D. H. Apaydin , M. C. Scharber , N. S. Sariciftci , ACS Appl. Energy Mater. 2020, 3, 10611. 33251486 10.1021/acsaem.0c01663 PMC7687026

[49] M. Jakešová , D. H. Apaydin , M. Sytnyk , K. Oppelt , W. Heiss , N. S. Sariciftci , E. D. Głowacki , Adv. Funct. Mater. 2016, 26, 5248.

[50] M. Gryszel , M. Sytnyk , M. Jakesova , G. Romanazzi , R. Gabrielsson , W. Heiss , E. D. Głowacki , ACS Appl. Mater. Interfaces 2018, 10, 13253. 29624365 10.1021/acsami.8b01295

[51] R. Wei , M. Gryszel , L. Migliaccio , E. D. Głowacki , J. Mater. Chem. C 2020, 8, 10897.

[52] M. Gryszel , R. Rybakiewicz , E. D. Głowacki , Adv. Sustainable Syst. 2019, 3, 1900027.

[53] N. Wadnerkar , V. Gueskine , E. D. Głowacki , I. Zozoulenko , J. Phys. Chem. A 2020, 124, 9605. 33166157 10.1021/acs.jpca.0c08496 PMC7681785

[54] M. Jakešová , M. S. Ejneby , V. Đerek , T. Schmidt , M. Gryszel , J. Brask , R. Schindl , D. T. Simon , M. Berggren , F. Elinder , E. D. Głowacki , Sci. Adv. 2019, 5, eaav5265. 30972364 10.1126/sciadv.aav5265 PMC6450690

[55] M. S. Ejneby , L. Migliaccio , M. Gicevic , M. Jakešová , F. Elinder , E. D. Głowacki , Adv. Mater. Technol. 2020, 5, 1900860.

[56] M. Gryszel , E. D. Głowacki , Chem. Commun. 2020, 56, 1705. 10.1039/c9cc09215c 31942910

[57] D. L. Wise , G. Houghton , Chem. Eng. Sci. 1966, 21, 999.

[58] S. A. M. van Stroe‐Biezen , F. M. Everaerts , L. J. J. Janssen , R. A. Tacken , Anal. Chim. Acta 1993, 273, 553.

