# Supplementary material for: Faradaic Pixels for Precise Hydrogen Peroxide Delivery to Control M‐Type Voltage‐Gated Potassium Channels
Source: Adv Sci (Weinh). 2021 Nov 26;9(3):2103132. doi: 10.1002/advs.202103132 (PMC8787424; doi:10.1002/advs.202103132)
Supplement: Supplementary file 1 — Supporting Information [file ADVS-9-2103132-s001.pdf]

ADVANCED SCIENCE
Open Access

Supporting Information

for *Adv. Sci.*, DOI: 10.1002/advs.202103132

Faradaic Pixels for Precise Hydrogen Peroxide Delivery to Control M-Type Voltage-Gated Potassium Channels

Oliya S. Abdullaeva[1,2], Ihor Sahalianov[1], Malin Silverå Ejneby[1,2], Marie Jakešová[3], Igor Zozoulenko[1], Sara I. Liin[4]*, Eric Daniel Głowacki[1,2,3]*

Supplementary information for:

Faradaic pixels for precise hydrogen peroxide delivery to control M-type voltage-gated potassium channels

Oliya S. Abdullaeva[1,2], Ihor Sahalianov[1], Malin Silverå Ejneby[1,2], Marie Jakešová[1,3], Igor Zozoulenko[1], Sara Liin[4]*, Eric Daniel Głowacki[1,2,3]*

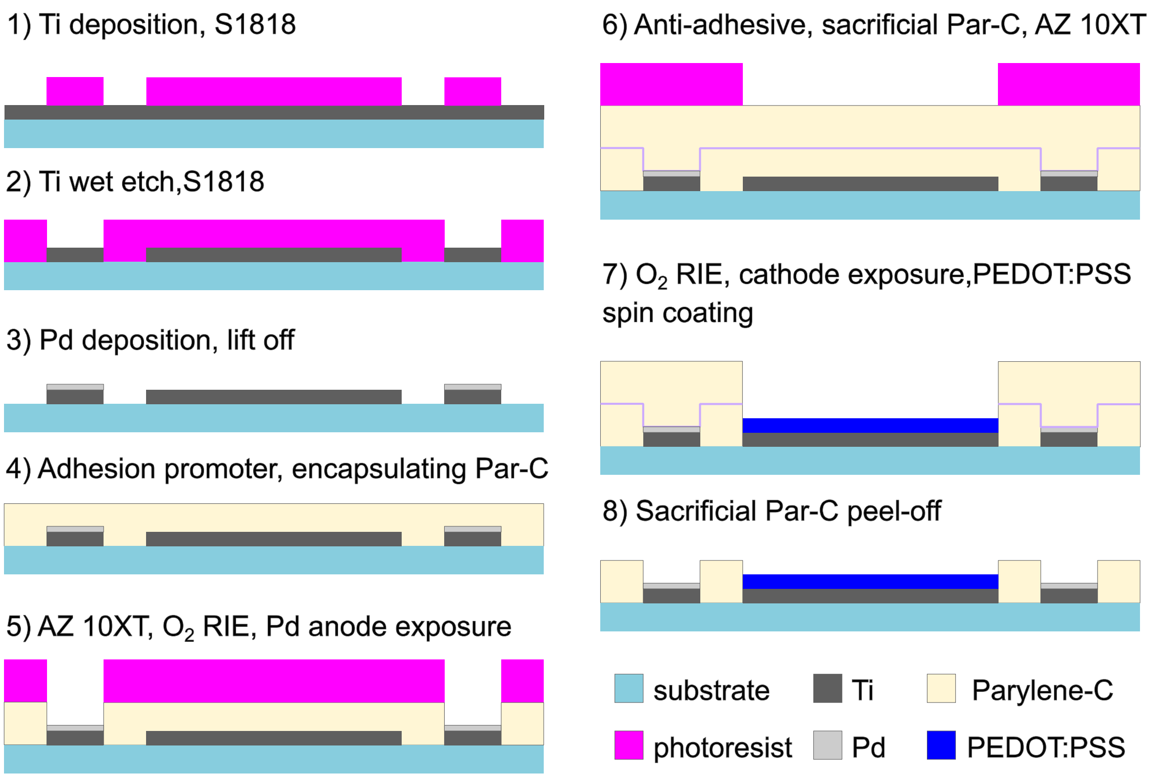

Fig. S1. Fabrication process flow for faradaic pixel devices. Metal layers are deposited via thermal evaporation, PEDOT:PSS is spin-coated from solution and patterned via parylene peel-off lithography.

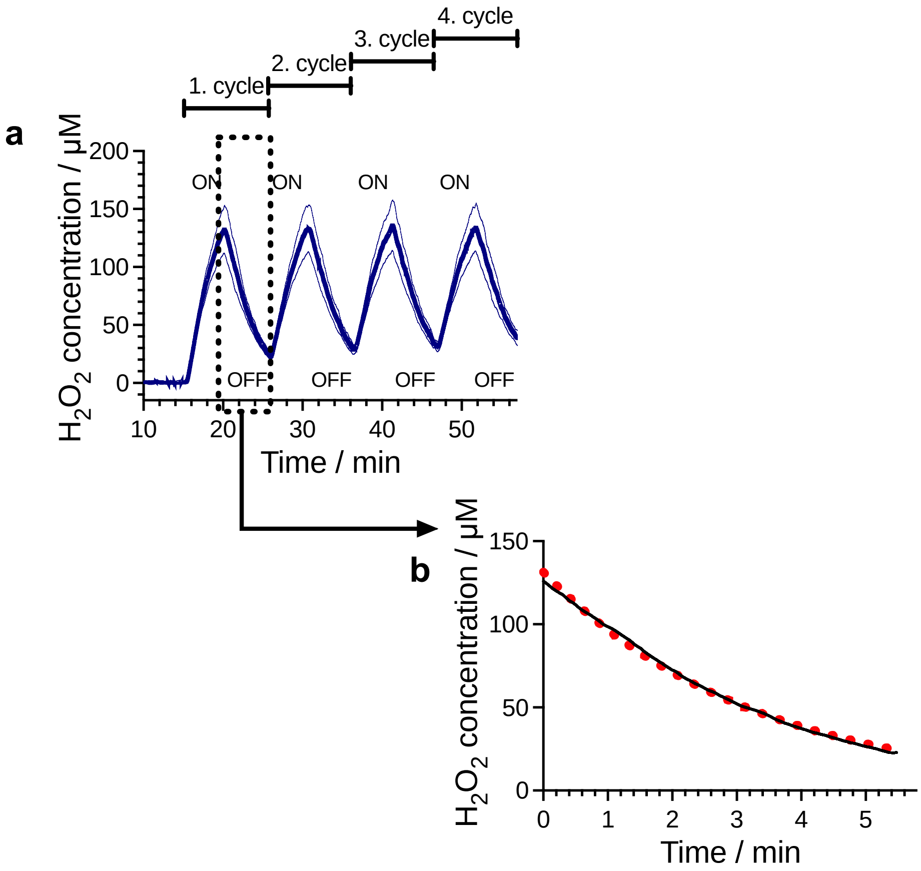

Fig. S2. Determination of half-life during *off*-period. Panel a shows time protocol with 4 consecutive *on*/*off* cycles and same data as in panel 1i. Panel b illustrates 1. *off*-period from panel a. The decay of the H_2O_2 concentration (black curve) is fitted with an exponential one phase decay function (red data points): Y = (Y_0 – Plateau) * exp(-K*X) + Plateau (X: Time, Y: H_2O_2 concentration which starts at Y_0 and decays (with one phase) down to Plateau, units of Y, Y_0 and Plateau: µM, K: rate constant equal to the reciprocal of the X axis units. Following constraints were applied: Plateau and K must be greater than 0. The half-life was found to be approx. 2.3 min for the 1. *off*-phase.

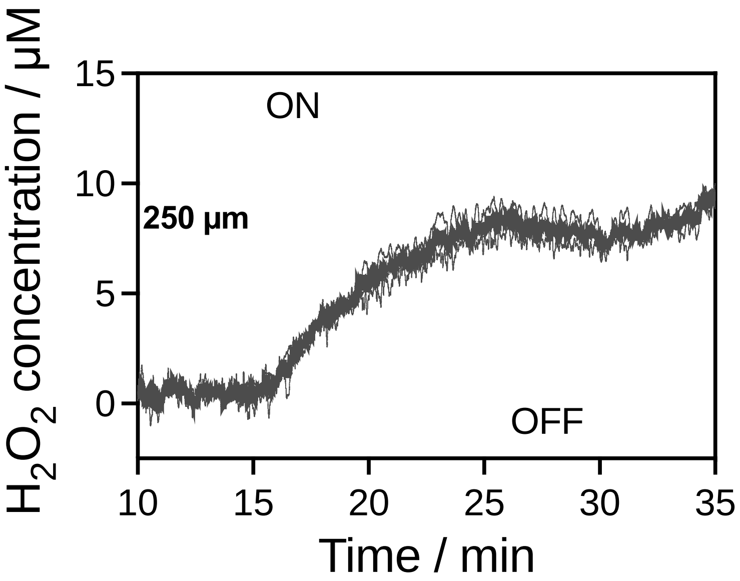

Fig. S3. Peroxide concentration registered over the palladium ring electrode. Due to oxidation and decomposition of peroxide at the palladium ring, the measured peroxide concentrations are more than ten times lower than at the region over the PEDOT cathode.

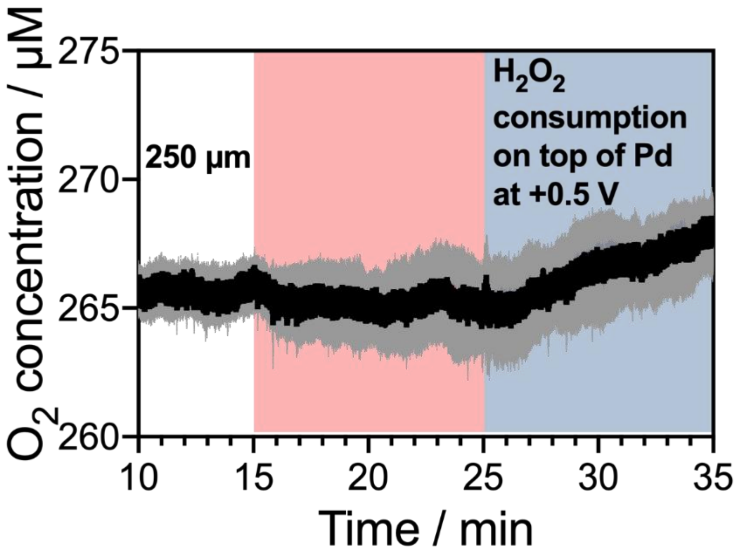

Fig. S4. Oxygen concentration recorded over the palladium ring electrode. After H_2O_2 generation via PEDOT cathode (15-25 minutes) only palladium ring electrode was operated in split configuration (25-35 minutes). The oxygen sensor was placed over the positively polarized palladium electrode (+0.5 V) to register increase in oxygen concentration upon H_2O_2 consumption (± SD, n=4).

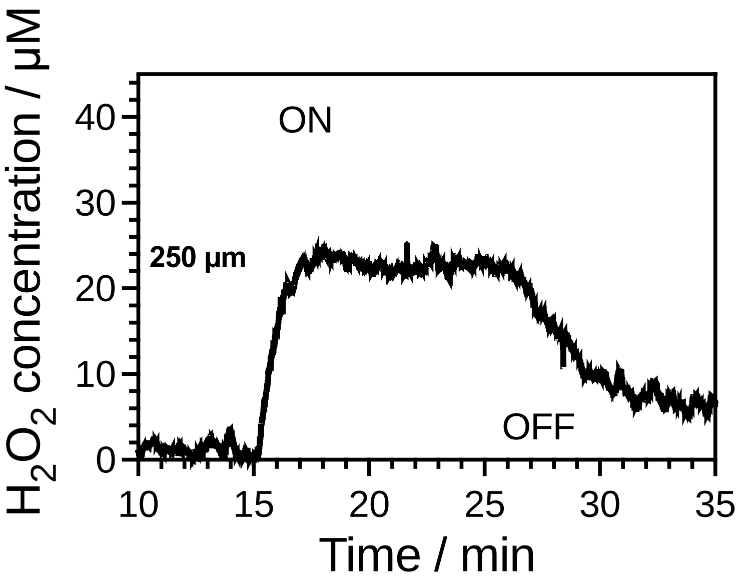

Fig. S5. H_2O_2 concentration recorded over a blank titanium electrode. During the *on*-period (15-25 minutes) the titanium back electrode was operated under galvanostatic condition (10 µA/cm^2) with *1K* covering both titanium and Pd counter electrode, similar to the operation conditions applied for the PEDOT faradaic pixel. Unlike with PEDOT, where peroxide concentration will increase to over 100 µM, titanium produces a much lower saturation value around 20 µM.

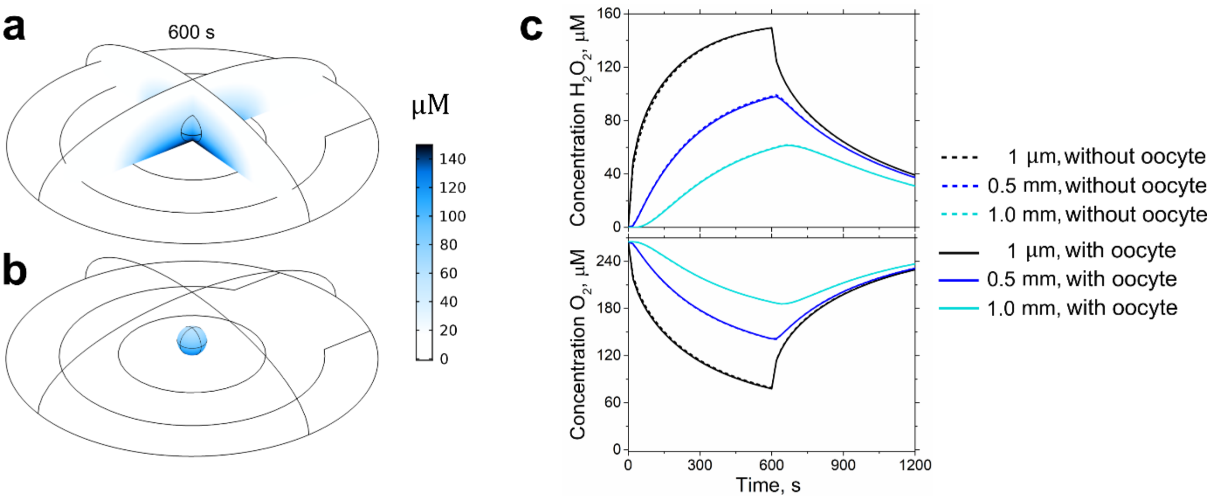

Fig. S6. Comparison of the calculated peroxide and oxygen concentrations both with and without oocyte included in the model. Panel **a,** H_2O_2 concentration profiles across the whole device with oocyte in the middle of PEDOT pixel. Panel **b**, H_2O_2 concentration depicted only on the surface of oocyte. Panel **c**, Comparing the concentrations of H_2O_2 at different distances from the center of PEDOT pixel with and without oocyte, included in the model. The cell membrane provides no effective barrier to the diffusion of oxygen or peroxide.

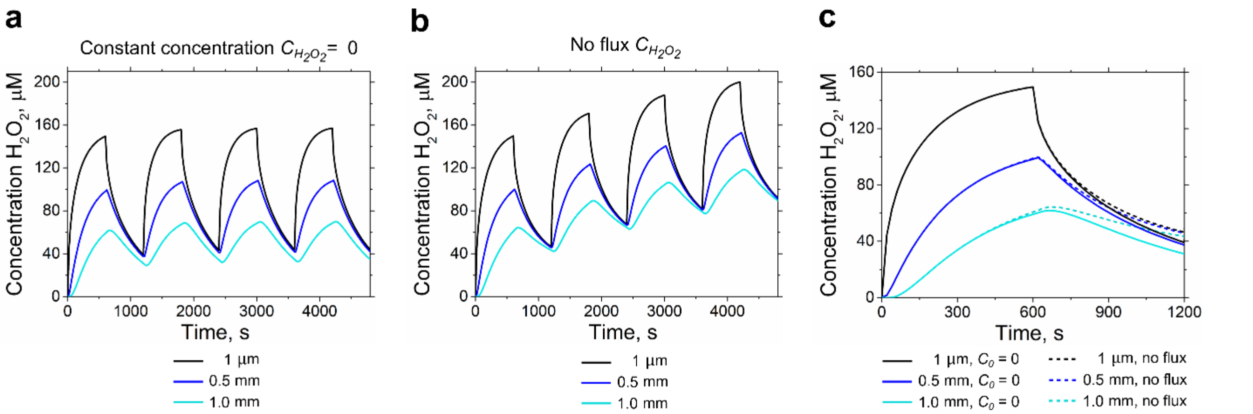

Fig. S7. H_2O_2 concentration profiles during four production cycles depending on boundary condition (BC) on top of the droplet. Panel **a**, Constant concentration BC $C_{H_2O_2} = 0$, H_2O_2 can diffuse from the droplet surface. Panel **b**, No flux BC, H_2O_2 cannot diffuse away from the droplet and all H_2O_2 consumption occurs on palladium region. The data simulated at constant concentration BC **a** agrees with the experimentally recorded values, while "no flux" BC **b** causes overestimation of H_2O_2 concentration with each cycle. Panel **c,** Data for both constant concentration and no flux BCs during the first cycle. Simulated H_2O_2 concentrations are the same during the first 600 s (production stage), but diverge during the following 600 s (consumption stage).

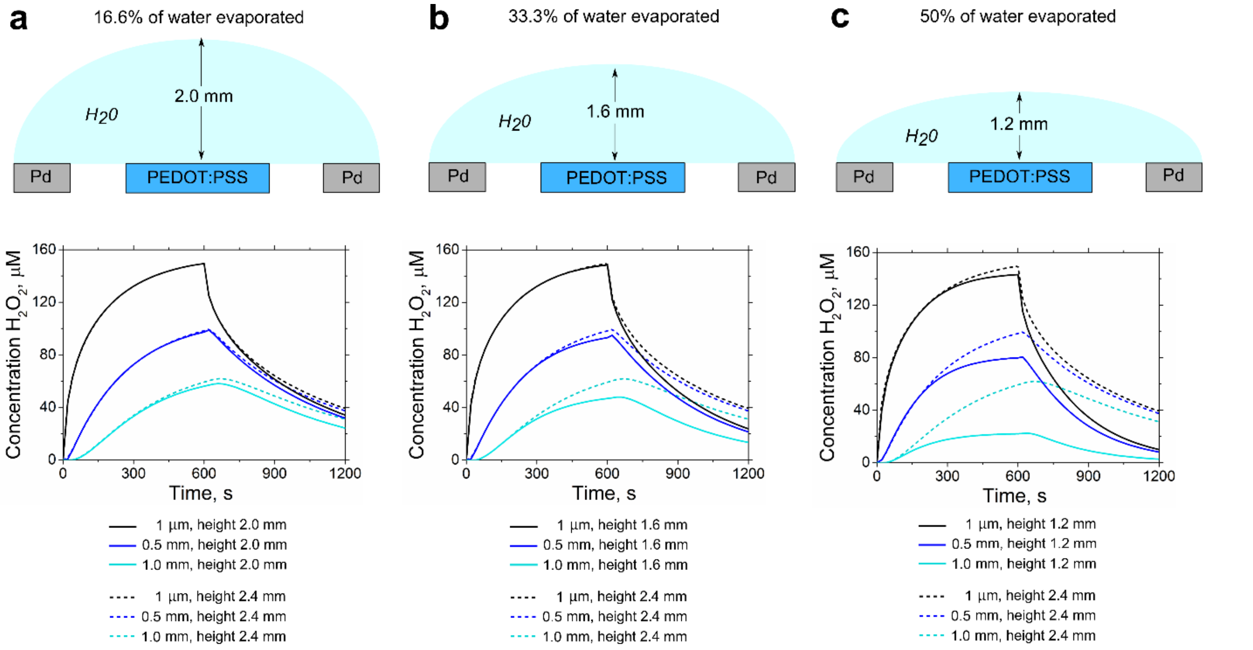

Fig. S8. The impact of droplet evaporation on H_2O_2 concentration gradients. Solid lines show concentrations of H_2O_2 in the initial droplet (200 microliters, 2.4 mm high), while dashed lines show concentrations in partially evaporated water droplets. The level of evaporation was varied by changing the droplet height from 2.4 mm to 1.2 mm, which corresponds to the evaporation of 50% of water. Evaporation of water leads to the faster dissipation of H_2O_2 on the distance from PEDOT pixel, while the concentration of H_2O_2 in the vicinity of PEDOT remains the same. At

significant levels of evaporation, the top side of an oocyte will be exposed to the significantly (approximately two times) smaller concentration of H_2O_2, while the concentrations of H_2O_2 near the medium and bottom parts of an oocyte will almost be the same, as in non-evaporated droplet.

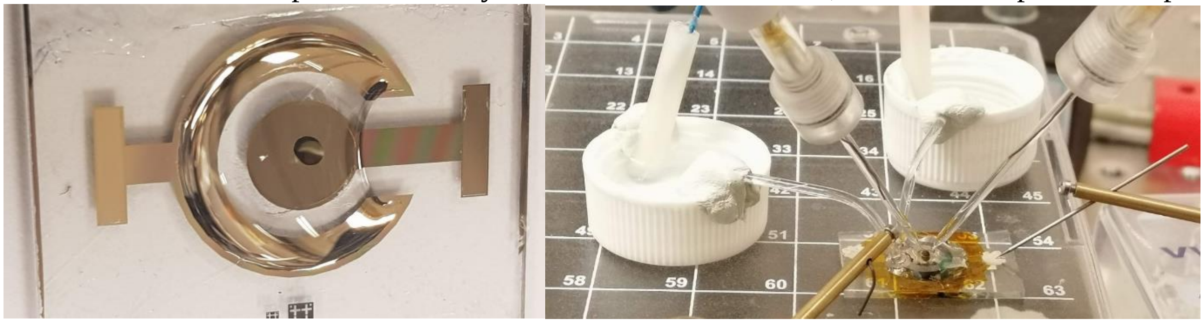

Fig. S9. TEVC measurement setup with oocyte positioned on the faradaic pixel device. Reference electrodes are separated from the droplet using salt bridges.

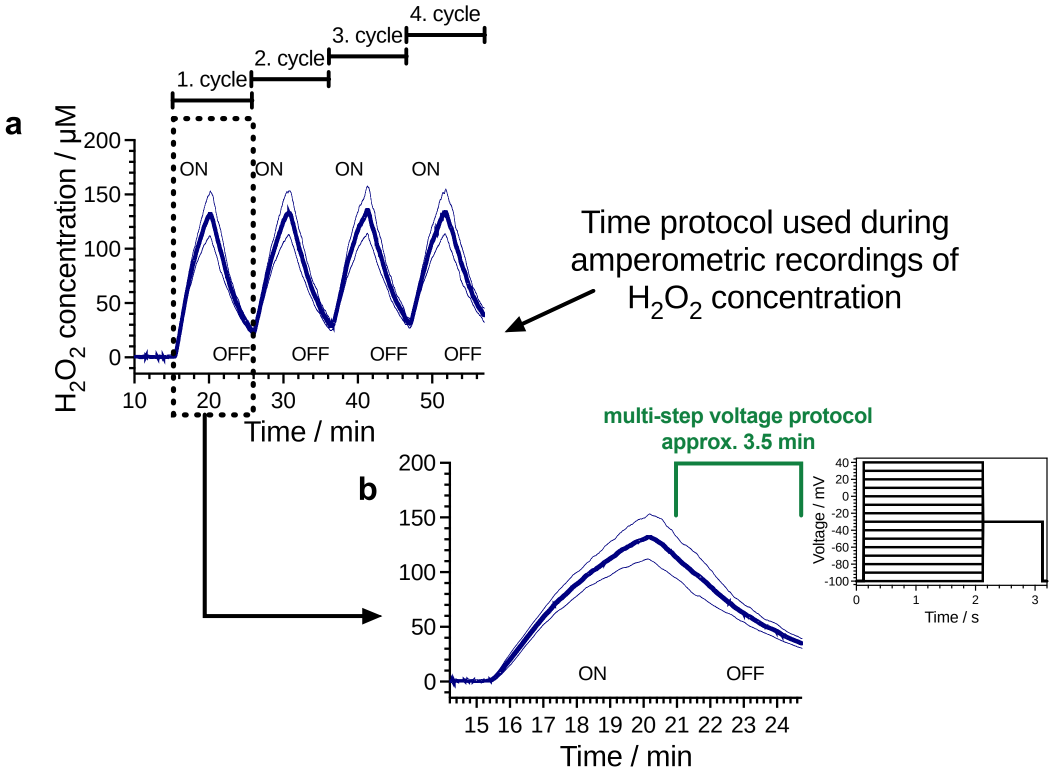

Fig. S10. Time protocol used during amperometric H_2O_2 measurements and electrophysiological recordings. Panel **a**, Same data as in panel 1i. The time protocol consists of a sequence of four *on/off* cycles. Panel **b** shows 1. cycle from panel a. Here, shaded area from 15-20 minutes indicates the period in which the faradaic pixel device is operated. The *on*-phase is followed by an *off*-period where the faradaic pixel is turned off for approx. 5.5 minutes. As highlighted by green lines the multi-step voltage protocol (inset panel b) is applied during the last 3.5 minutes of the *off*-period. To obtain I_{ss} and I_{tail} in time-match control recordings the same time

protocol was used by applying the multi-step voltage protocol during each *off*-period without operating the PEDOT cathode in the prior *on*-phase.

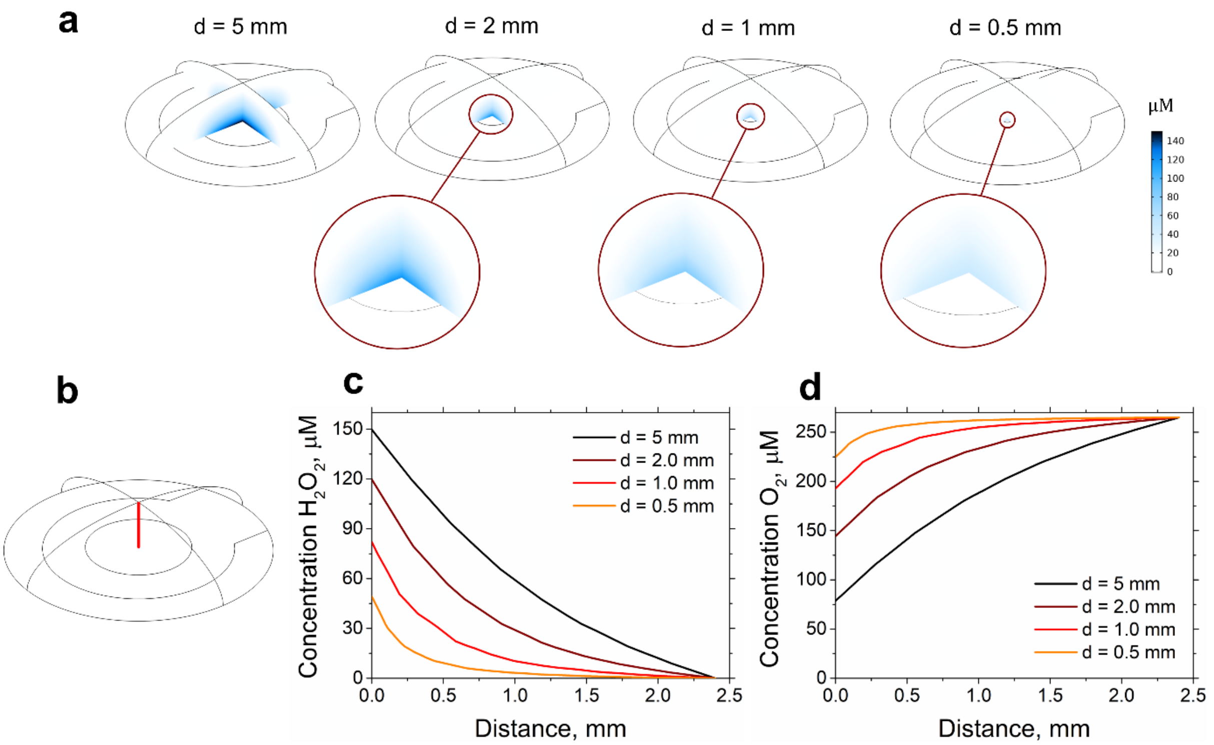

Fig. S11. H_2O_2 and O_2 production/consumption for PEDOT pixels of different diameters and same current densities. Panel **a**, H_2O_2 concentration profiles for pixel diameters from 5 mm to 0.5 mm, insets show zoomed profiles near pixels. Panel **b**, Line segment (highlighted in red) connecting the center of PEDOT pixel and the top of a water droplet. Panel **c-d**, H_2O_2 and O_2 concentrations, extracted along red line segment depicted in **b** for different PEDOT pixel diameters. Downsizing of PEDOT pixel diameter leads to the increase of H_2O_2 concentration gradient. A high local concentration of peroxide is observed at the production area for small PEDOT pixels, with a rapid decrease towards the droplet surface. Also, small PEDOT pixels prevent oxygen depletion because of lesser O_2 consumption and fast diffusive compensation from the outer droplet region.
