# Supplementary figures and images for: Faradaic Pixels for Precise Hydrogen Peroxide Delivery to Control M‐Type Voltage‐Gated Potassium Channels
Source: Adv Sci (Weinh). 2021 Nov 26;9(3):2103132. doi: 10.1002/advs.202103132 (PMC8787424; doi:10.1002/advs.202103132)

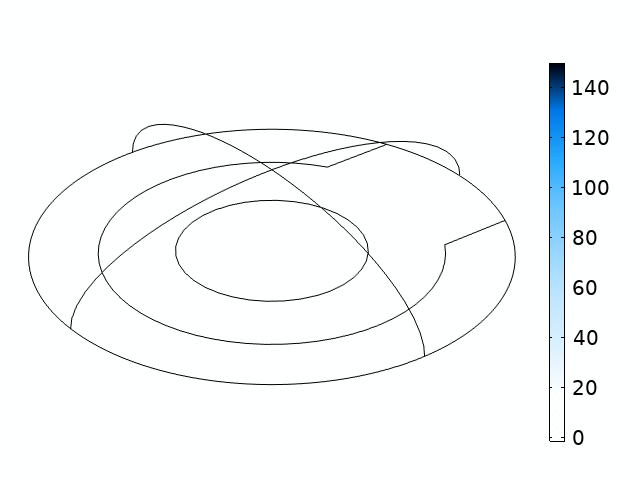

Supplement: Supplementary file 2 — Supplemental Video 1 [file ADVS-9-2103132-s002.gif]
